# Methylglyoxal: a novel upstream regulator of DNA methylation

**DOI:** 10.1186/s13046-023-02637-w

**Published:** 2023-03-31

**Authors:** Gaurav Dube, Assia Tiamiou, Martin Bizet, Yasmine Boumahd, Imène Gasmi, Rebekah Crake, Justine Bellier, Marie-Julie Nokin, Emilie Calonne, Rachel Deplus, Tom Wissocq, Olivier Peulen, Vincent Castronovo, François Fuks, Akeila Bellahcène

**Affiliations:** 1 Laboratory of Cancer Epigenetics, Faculty of Medicine, ULB-Cancer Research Center (U-CRC), Université Libre de Bruxelles (ULB), Brussels, Belgium; 2 Metastasis Research Laboratory, GIGA-Cancer, GIGA Institute, University of Liège, Liège, Belgium; 3 WELBIO (Walloon Excellence in Lifesciences & Biotechnology), Brussels, Belgium

**Keywords:** Methylglyoxal, DNA methylation, Breast cancer, Tumor suppressor genes, Metastasis

## Abstract

**Background:**

Aerobic glycolysis, also known as the Warburg effect, is predominantly upregulated in a variety of solid tumors, including breast cancer. We have previously reported that methylglyoxal (MG), a very reactive by-product of glycolysis, unexpectedly enhanced the metastatic potential in triple negative breast cancer (TNBC) cells. MG and MG-derived glycation products have been associated with various diseases, such as diabetes, neurodegenerative disorders, and cancer. Glyoxalase 1 (GLO1) exerts an anti-glycation defense by detoxifying MG to D-lactate.

**Methods:**

Here, we used our validated model consisting of stable GLO1 depletion to induce MG stress in TNBC cells. Using genome-scale DNA methylation analysis, we report that this condition resulted in DNA hypermethylation in TNBC cells and xenografts.

**Results:**

GLO1-depleted breast cancer cells showed elevated expression of DNMT3B methyltransferase and significant loss of metastasis-related tumor suppressor genes, as assessed using integrated analysis of methylome and transcriptome data. Interestingly, MG scavengers revealed to be as potent as typical DNA demethylating agents at triggering the re-expression of representative silenced genes. Importantly, we delineated an epigenomic MG signature that effectively stratified TNBC patients based on survival.

**Conclusion:**

This study emphasizes the importance of MG oncometabolite, occurring downstream of the Warburg effect, as a novel epigenetic regulator and proposes MG scavengers to reverse altered patterns of gene expression in TNBC.

**Supplementary Information:**

The online version contains supplementary material available at 10.1186/s13046-023-02637-w.

## Background

In last decades, major features of aggressive tumors such as metastatic expansion and drug resistance have been related to metabolic reprogramming. The best-studied metabolic property observed in cancer cells is indisputably the Warburg effect. Contrarily to normal cells, most of cancer cells rely on glycolysis for building biomass to sustain their proliferation and ATP production, even in presence of oxygen [1]. A less understood and explored facet of this process is its significant role in the production of methylglyoxal (MG), a highly reactive dicarbonyl molecule. In glycolytic cancer cells, MG is mainly formed from the spontaneous conversion of dihydroxyacetone phosphate and glyceraldehyde 3-phosphate along the glycolysis pathway [2]. Cellular MG concentration is notably regulated by the detoxifying activity of the glyoxalases system, with glyoxalase 1 (GLO1) being the rate-limiting enzyme [3]. We previously used GLO1 stable depletion strategy in MDA-MB-231 TNBC breast cancer cells to induce endogenous MG accumulation and the formation of glycated adducts resulting in cellular dysfunction, hereafter mentioned as MG stress [4, 5]. We showed that GLO1-depleted MDA-MB-231 cells acquired enhanced growth and metastatic capacity in vivo that could be efficiently reverted using carnosine, a natural dipeptide with potent MG scavenging capacity [4]. Our transcriptomic study of cancer cells with low MG detoxification capacity notably pointed to the regulation of MAPK signaling, extracellular matrix remodeling and cell migration processes [6].

MG is a very reactive glycating agent of proteins, lipids and DNA to form advanced glycation end products (AGEs) [7, 8]. For non-enzymatic glycation of proteins, MG primarily reacts with arginine residues to form MG-derived hydroimidazolone [9] and argpyrimidine (ArgPyr) [10] adducts. We demonstrated that ArgPyr accumulation is a common feature in mammary [11] and colorectal [12, 13] malignant tumors when compared with their normal counterparts. Identification of MG-modified proteins remains a challenge in glycation research. A short list of well-studied susceptible targets comprises extracellular proteins such as albumin [14], hemoglobin [15] and type IV collagen [16]. In cancer cells, we and others contributed to demonstrate that heat shock proteins (HSPs) are key MG targets. When modified by MG, HSP27 favored cancer cell escape from apoptosis [17–19] and HSP90 induced the blockade of Hippo tumor suppressor pathway [4]. More recent studies pointed to intense MG glycation on histones under basal and high glucose conditions across normal and cancer cell lines [20] and in breast tumors [21].

Next to histone code, epigenetic regulation of gene expression is governed by DNA methylation. In cancer, specific hot spots of hypermethylated CpGs are concentrated at gene promoter and enhancer regulatory domains [22]. Lately, DNA methylation at enhancers gained growing interest and notably pinpointed differentially methylated enhancers between ER-positive and ER-negative tumors [23]. DNA methyltransferases (DNMTs), responsible for DNA methylation, inhibit the expression of tumor suppressor genes (TSGs), conferring growth, invasion and survival advantages to cancer cells [24]. Accordingly, the cytosine analogue 5-Aza-2’-Deoxycytidine (5-AZA) reactivates silenced TSGs and has therapeutic efficacy in hematopoietic malignancies [25] and in selected solid tumors [26]. When compared with other breast cancer subtypes, TNBC showed extensive hypermethylation of specific epigenetically regulated genes [27]. Up-regulation of the expression and activity of DNMTs, and particularly DNMT3B, has been associated with the acquisition of a hypermethylator signature in breast cancer [28].

Independently of one another, metabolic reprogramming and DNA hypermethylation aberrations have been extensively studied in breast cancer. However, the possibility that a glycolysis by-product could be the trigger of major epigenetic changes on DNA has never been investigated to date. In this study, we used genome scale methylation analysis combined with transcriptome profiling to show that the pro-cancer effects of MG accumulation are associated, at least in part, with extensive DNA hypermethylation notably leading to the silencing of specific metastasis-related TSGs. These latter delineated a unique MG signature capable of segregating TNBC patients with poor prognosis. Importantly, our findings position MG stress as a druggable target upstream of DNA methylation machinery in breast cancer.

## Methods

### Cell culture and reagents

MDA-MB-231 and Hs578T TNBC breast cancer cell lines were obtained from the American Type Culture Collection (ATCC, Manassas, VA, USA). Cells were cultured in Dulbecco’s modified Eagle’s medium (DMEM) (Lonza) containing 10% fetal bovine serum (FBS, ThermoFisher Scientific) and 2 mM L-glutamine (Lonza), unless otherwise specified. L-carnosine (C9625), aminoguanidine (396,494), methylglyoxal (MG, M0252) and 5’Aza-2’-Deoxycytidine (A3656) and cycloheximide (C7698) were from Sigma.

### shRNA transduction

We previously described the generation and the characterization of shGLO1 and shNT stable clones [4]. Briefly, we used commercially available shRNAs targeting GLO1 (shGLO1) and non-target (shNT) (shGLO1#1, TRCN0000118627) and shGLO1#2, TRCN0000118631) and non-target NT, anti-eGFP shRNA, SHC005 (all from Sigma-Aldrich) and shRNA transfection using lentiviral vectors was performed at the GIGA Institute Viral Vectors platform (University of Liège). Transduced MDA-MB-231 and Hs578T cells were selected with 1 µg/ml and 0.5 µg/ml of puromycin (P9620, Sigma-Aldrich), respectively.

### DNA methylation data analysis

#### Sample processing

We used MDA-MB-231 breast cancer cells, 3 biological replicates per condition (shNT-1, -2 and -3, shGLO#1–1, -2 and -3 and shGLO1#2–1, -2 and -3) and MDA-MB-231 mouse tumor xenografts, 3 mice per condition (shNT and shGLO1#2). Genomic DNA was extracted with the QIAamp DNA Mini Kit (Qiagen, Hilden, Germany) as previously described [29]. Genomic DNA (500 ng) was converted with sodium bisulfite using the Zymo EZ DNA Methylation Kit (Zymo Research, Orange, USA) following the alternative incubation conditions of the manufacturer recommended for Illumina Infinium Human Methylation assays. Using 4 µl converted DNA at 50 ng/µl according to the manufacturer’s protocol, DNA methylation was assessed on Infinium HumanMethylation 850 K bead-arrays. Illuminaio R package was used to extract raw probe intensity values (raw data). The quality of array data was evaluated visually by assessing the intensity level of the control probes. All samples that showed the expected profiles for the different control probes were utilized for further analyses.

#### Infinium Human Methylation 850K data pre-processing

Raw data (uncorrected probe intensity values) from the Infinium Methylation arrays were processed with the following steps: probes of low quality (detection p-value threshold > 0.05), cross-reactive probes (*i.e.* targeting several genomic locations) as well as probes containing SNPs based on the extended annotation of Mc Cartney et al. [30] were removed. Additionally, probes targeting X and Y-chromosomes were also removed from the analysis. Beta-values were computed using the formula: β-value = M / [U + M], where M and U are the raw “methylated” and “unmethylated” signals, respectively. Beta-values were corrected for type I and type II bias using the peak-based correction [31].

#### Infinium Human Methylation 850K annotation

To define promoter, enhancer and gene body, we used the re-annotation of the EPIC array that we have recently published [32]. Briefly, the promoter and enhancer were defined using ENCODE genome segmentation, which is based on histone marks. The promoter of any gene is therefore defined as the promoter region overlapping with the transcription start site (TSS) of that gene. Enhancers were associated to gene using the EnhancerAtlas database (*i.e.* any ENCODE-defined enhancer region also present in the EnhancerAtlas database is associated to the gene predicted to be its target by the database). Finally, gene body were defined as region between TSS and transcription terminal site (TTS) of a gene.

#### Differential methylation analysis

To identify differential CpGs between shNT and shGLO1 condition we followed recommendations from [31]. First, the methylation values were converted to M-values using the following formula: M-value = log2 (β-value/(1–β-value)). Then the statistical significance of the differential methylation was assessed using a t-test applied on these M-values. P-values were corrected for multi-testing using Benjamini–Hochberg method. In parallel, median β-values _(shNT)_ for each CpG from three control replicates and median β-values _(shGLO1)_ from six shGLO1 replicates were calculated. Then median Δβ was calculated using formula: Δβ = median CpG β-value _(shGLO1)_ – median CpG β-value _(control)_. CpGs with Δβ value above 0.2 and corrected p-value less than < 0.05 was considered as differentially hypermethylated CpGs, while lower than -0.2 as differentially hypomethylated.

### ROS measurement by FACS

ROS production was measured using CM-H2DCFDA fluorescent probe (Invitrogen) according to the manufacturer’s protocol. Briefly, MDA-MB-231 cells cultured in low- (1 g/L) or high-glucose (4.5 g/L) medium and GLO1-depleted cells were trypsinized and incubated with the probe (diluted 1/5000 in HBSS) for 15 min in the dark. After centrifugation, cells were incubated in their corresponding culture medium during 15 min at 37 °C before FACS analysis. H_2_0_2_ treatment (100 μΜ) was used as positive control for ROS induction.

### Cellular MG quantification

MBo (Methyl diaminobenzene-BODIPY) was used to specifically detect endogenous MG [33]. MDA-MB-231 cells cultured in low- (1 g/L) or high-glucose (4.5 g/L) medium were treated with 5 mM MBo. After 1 h, the cells were washed with PBS and incubated in the corresponding culture medium for 6 h. Cells were then trypsinized and analyzed by flow cytometry (FACSCanto, BD Biosciences).

### Pathway analysis of methylation data

List of genes corresponding to differentially methylated CpGs (between shNT and shGLO1) was ranked according to their Δβ (rank file) and a GMT file composed of C6 oncogenic signature gene sets [34] manually curated and reclassified into oncogene inhibition (OG-I) and tumor suppressor gene activation (TSG-A) signatures (Data S2) was submitted to Gene Set Enrichment Analysis (GSEA) tool [35]. Signatures identified with *p*-values and FDR q-values less than 0.05 were called significant and positive normalized enrichment score (NES) considered further.

### Analysis of gene expression data

We analyzed the previously published RNAseq data [6] of MDA-MB-231 GLO1-depleted cells along with their controls using Kallisto—Sleuth pipeline. Quality control and preprocessing of reads was performed using FastQC (v0.11.4) (http://www.bioinformatics.babraham.ac.uk/projects/fastqc/) and Trimmomatic (v0.36) [36]. Kallisto (v0.44.0) package [37] was utilized for indexing the ENSEMBL cDNA transcripts (Human assembly hg38 (GRCh38), ENSEMBL release 92) using the “index” function and quantifying RNA abundance, using “quant” function, with a number of bootstrap set to 100, at gene- as well as transcript-level in transcripts per million (TPM) counts.Differential gene expression was performed using Sleuth R package (v0.30.0) [38] to benefit from the bootstrap estimates of Kallisto and yield a gene-level normalized TPM matrix. For each gene, both the likelihood ratio test (LRT) and Wald test (WT) were applied on the condition parameter to obtain their respective False Discovery Rate (FDR)-corrected p-values. Significantly expressed genes were those passing the two tests at a cutoff of FDR < 0.05. The beta value generated from the Wald test was used as synonym to the fold change between shGLO1 and shNT conditions.

### Bisulfite pyrosequencing validation

Confirmation of Infinium data was performed using bisulfite genomic pyrosequencing. Converted DNA was used as template in each subsequent PCR. Primers for PCR amplification and sequencing were deduced with the PyroMark® Assay Design 2.0 software (Qiagen) and are listed in Table S1. PCRs were performed with the HotStarTaq DNA polymerase PCR kit (Qiagen) under the following conditions: 95 °C 15 min; 50 cycles of [95 °C 30 s; Tm 1 min; 72 °C 1 min]; 72 °C 7 min. Some genes required a nested PCR, for which 10 µl of the first amplification were engaged in a second one using the same time parameters (see Table S1). The success of amplification was assessed by agarose gel electrophoresis and the pyrosequencing of the PCR products was performed with the Pyromark™ Q24 system (Qiagen).

### Immunoblotting

Cells were extracted with SDS 1% buffer containing proteases and phosphatases inhibitors (Roche). Protein extracts were loaded in 7.5%, 10% or a 12.5% gel and transferred to a PVDF membrane (Roche). After 1 h of blocking with 5% non-fat milk (Biorad) in Tris Buffered saline- 0.1% Tween 20 (TBS-T), membranes were incubated with the indicated primary antibodies (Table S2) overnight at 4 °C. After washes in TBS-T, membranes were incubated during 1 h with the corresponding secondary antibodies. Chemiluminescent detection of protein expression was performed using Pierce ECL Western (BioRad) or Clarity™ Western ECL Substrate (BioRad) depending on the expression level of proteins of interest. Band densities were quantified with Image J Software (https://imagej.nih.gov/ij/).

### RNA isolation and reverse transcription- quantitative PCR (RT-QPCR)

RNA extraction was performed according to the manufacturer’s protocol (NucleoSpin RNA, Macherey–Nagel, Düren, Germany). The reverse transcription was achieved with 2 µg of total RNA mixed with random hexamer primers (Thermoscientific), dNTP mix (Thermoscientific), RiboLock RNAse inhibitor (Thermoscientific), reaction Buffer (Promega) and M-MLTV reverse transcriptase (Promega). For RT-QPCR, 100 ng of cDNA were mixed with primers, probe (Universal ProbeLibrary System, Roche) (Table S3) and 2 × Takyon Rox Probe MasterMix dTTP Blue (Eurogentec, Seraing, Belgium). Q-PCR were performed under the following conditions: 95 °C 10 min; 40 cycles of [95 °C 15 s; 60 °C 1 min] using the QuantStudio™ 3 Real-Time PCR Systems using the corresponding manufacturer’s software (Applied Biosystems, Carlsbad, CA). The expression of genes of interest was normalized to 18S rRNA to obtain a relative mRNA expression in 2^−ΔΔCt^.

### Real-time cell migration assay

Cells were seeded in a 6–well plate and treated with 5-AZA or transfected with siRNAs specifically directed against DNMT3B. Eighty thousand cells from the 6-well plates were seeded in IncuCyte® ImageLock 96-well microplates and were scratched with the woundmaker scratcher. Then, cells were washed with the appropriate medium and treated again with 5-AZA when indicated. The monitoring and the analysis of the migration process were performed using the IncuCyte S3 System. The percentage of relative wound closure was calculated as follows: [(Wound width time 0 – Wound width time 1) / (wound width time 0)] * 100.

### Xenografts

Tumor material from MDA-MB-231 cells xenografted in mice was available from our previous study [4]. Briefly, MDA-MB-231 shNT and shGLO1#2 cells were suspended in 10% FBS culture medium and Matrigel (BD Biosciences) (1:1 v/v). One million of cells were subcutaneously inoculated in one flank of 5-week-old female NOD-SCID mice. Tumors were collected and frozen in liquid nitrogen until DNA, protein or RNA extraction, as indicated.

### Meta integration of differential methylation, gene expression and pathway data

The data from differential gene expression and differential methylation analysis mentioned above were merged at gene-level using in-house R script. Genes having differentially up-regulated expression and hypomethylation were retrieved and called as differential gene list 1 (Data S4); and those having differentially down-regulated expression and hypermethylation were called as differential gene list 2 (Data S3). As we understand from the literature [39] that hypermethylation in the promoter or enhancer region of a gene causes its down-expression, we considered methylation of only promoter or enhancer for retrieving the above differential gene lists (Fig. 3A). As the scope of our work is oriented towards hypermethylation and down-expression of genes caused by MG stress, we focused our attention on list 2 genes (Data S3) and used them for all the following analysis. For the sake of completeness, genes having differentially up-regulated expression and hypermethylation in gene body are listed in Data S5; and those having differentially down-regulated expression and hypomethylation in gene body are listed in Data S6.

Additionally, as depicted in Fig. 3A, results from the GSEA pathway analysis of methylation and gene expression data were integrated. Statistically significant pathways (*p*-values < 0.05, FDR q-value < 0.05) with positive NES from methylation and expression results were overlapped and called as ‘epigenetically repressed pathways’ (Fig. 3A). Common genes composing these pathways were identified and further overlapped with list of ‘epigenetically repressed genes’ to yield a ‘60-gene MG signature’.

As this ‘60-gene MG signature’ was derived from cell line data, we next aimed at generating a clinically relevant TNBC-specific signature using METABRIC breast cancer patients data [40, 41]. For this purpose, signature optimization was carried out using pathway correction method where expression values of each gene were correlated with their respective pathway module score (see score calculation formula below) and genes with correlation R > 0.25 and p-value < 0.05 were kept. This resulted in a refined and clinically relevant ‘14-gene MG signature’ that was further correlated with clinical parameters and other specific metabolic signatures.

### MG score calculation

A score corresponding to MG signature (*i.e.* MG score) and other signatures used in this work was calculated using the following R function:

$$\begin{aligned}signature & \_score <- function(gene\_list, expr\_df)\{\\ & expr\_df <- expr\_df[intersect(gene\_list, rownames(expr\_df)),]\\ & expr\_df <- as.matrix(expr\_df)\\ & scaled\_expr\_df <- apply(expr\_df, 1, scale)\\ & scaled\_expr\_df <- t(scaled\_expr\_df)\\ & colnames(scaled\_expr\_df) <- colnames(expr\_df)\\ & score <- apply(scaled\_expr\_df, 2, mean, na.rm=TRUE)\\ & return(-1*score)\}\end{aligned}$$

where gene_list is a character vector of genes in the signature module and expr_data is a numeric dataframe with rows as genes and columns as samples. The ‘signature score’ is computed as the z-score normalized-expression sum for the signature genes: each gene expression is first z-score normalized across patients (*i.e.* we set the mean expression of each gene to zero and its expression value for each patient is then represent as its number of standard deviation toward the mean). This step allows each gene to contribute equally to the score. Then the signature_score is computed, for each patient, as the mean of the normalized expression of all the signature genes. For a more intuitive score, the final value was multiplied by -1 thus allowing the score to correlate with the expected MG stress level.

### Statistical analysis

Experimental data from two groups were compared with unpaired t-test and experimental data from more than two groups were compared using one-way or two-way analysis of variance (ANOVA) depending on the number of grouping factors. Dunnett’s test was applied for multiple comparisons. A bilateral *p* value < 0.05 was considered as statistically significant. Kaplan–Meier survival curves with log-rank tests, recording patients at the time of death or disease recurrence or last follow-up visit, were used to compare overall survival or disease-free survival rates. All p-values were two-sided, and *p*-values of less than 0.05 were considered statistically significant.

### Raw data availability

The Infinium DNA methylation data related to the study are publicly available in the NCBI’s GEO database (GEO ID: GSE185237).

## Results

### Knockdown of GLO1 contributes to major changes in DNA methylation in breast cancer cells

To interrogate DNA methylation status under low MG detoxification condition, we performed methylation profiling using Infinium 850K human methylation array on MDA-MB-231 TNBC cells stably depleted for GLO1 and on control cells (referred hereafter as shGLO1 and shNT, respectively). Efficient GLO1 depletion at the protein level in MDA-MB-231 cells is shown in Fig. S1A. Elevated levels of MG and its protein adducts in shGLO1 MDA-MB-231 cells have been validated in a previous study [4]. For the first time, our methylome analysis pointed to major alterations in the epigenome associated with decreased GLO1-mediated detoxification in breast cancer cells. Principal component analysis (PCA) of DNA methylation data based on the 2000 probes showing the highest standard deviation emphasized a broad pattern of methylation in GLO1-depleted cells that was clearly different from shNT control cells (Fig. S1B). Assuring reliability of DNA methylation data, β-values of selected representative genes obtained using pyrosequencing technique showed consistent correlation with Infinium array methylation data (Pearson correlation coefficient R = 0.955, *p*-value = 7.23e-72, Fig. S1C).

**Fig. 1:**
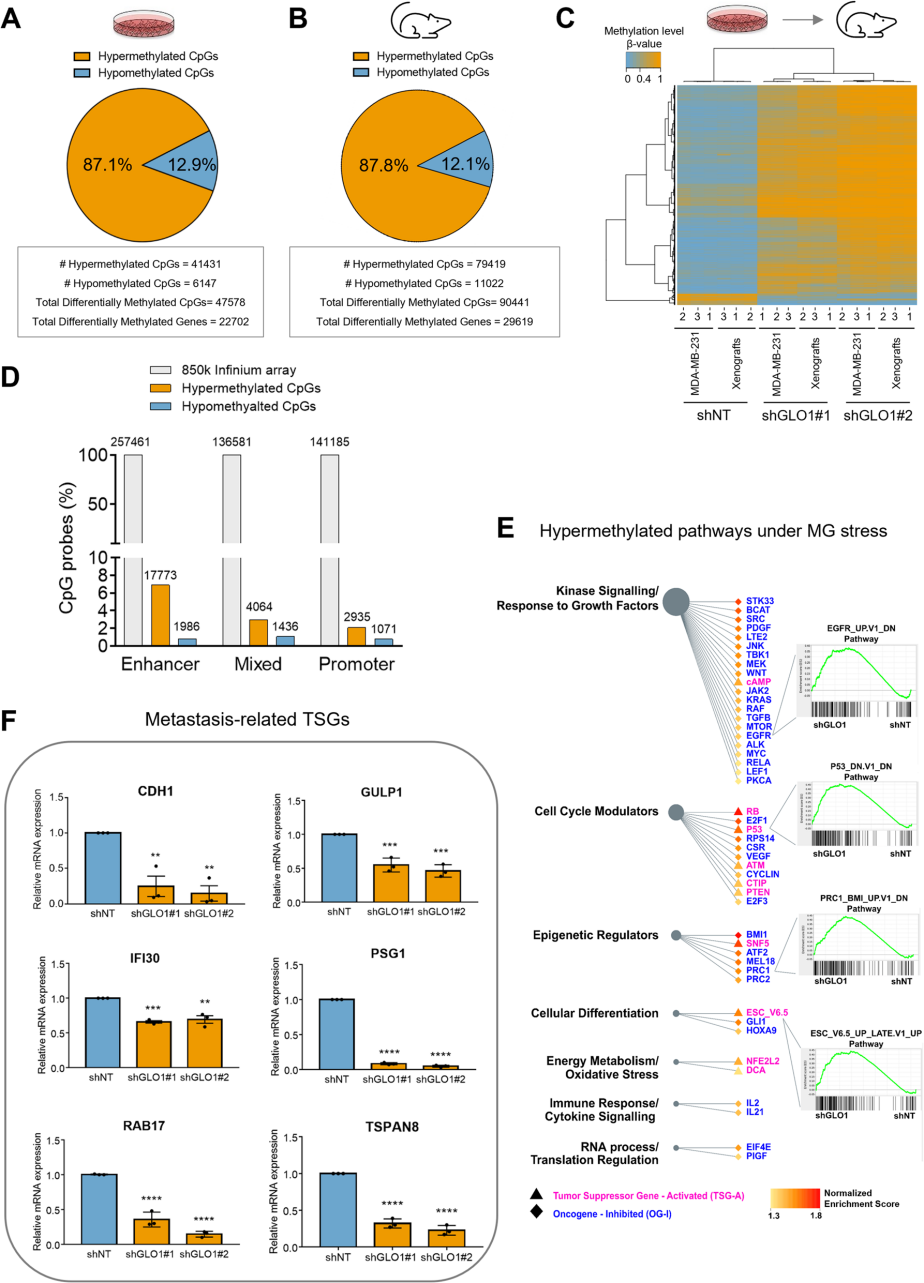
GLO1-depleted MDA-MB-231 breast cancer cells exhibit major DNA hypermethylation and loss of metastasis-related TSGs. **A** and** B **Pie charts summarizing the proportion of hypomethylation (FDR < 0.05, Δβ < -0.2) and hypermethylation (FDR < 0.05, Δβ > 0.2) within differentially methylated CpGs (DMCs) found in MDA-MB-231 cells and mouse xenografts, respectively. **C **Heatmap representing unsupervised clustering of DMCs (rows) identified between control (shNT, *n* = 3) and GLO1-depleted (shGLO1, *n* = 6) cells (columns) and their corresponding status in xenograft methylation data. Color key scale blue: low methylation and orange: high methylation. **D** Proportion of hypo- and hypermethylated DMCs distributed across the genome regulatory regions. Mixed regions correspond to Infinium array probes referring to either promoter or enhancer, according to the considered cell line. **E** Tumor suppressor gene activated (TSG-A) and oncogene inhibited (OG-I) pathways enriched in genes that were affected by hypermethylation in GLO1-depleted cells as estimated from GSEA tool enrichment scores (with FDR < 0.05). For example, P53 knockdown led to down expression of genes that composed the P53 TSG pathway (‘P53_DN.V1_DN’) whose activation (TSG-A) was affected by high methylation (enrichment score). Please refer to Data S8 for more details on TSG-A and OG-I pathways. **F** Representative metastasis-related TSGs that were hypermethylated and low expressed under MG stress. Data represent the mean values ± SEM of three independent experiments and were analyzed using a one-way analysis of variance (ANOVA) followed by a Dunnett test (** *p* < 0.01, *** *p* < 0.001 and **** *p* < 0.0001)

By comparing control and GLO1-depleted cells, we identified 47,578 differentially methylated CpGs (DMCs) accounting for 22,702 genes, among which the large majority (41,431 DMCs; 87.1%) was hypermethylated in shGLO1 cells (Fig. 1A, Data S1) indicating a significant expansion of genome methylation upon GLO1 depletion in breast cancer cells. These 41,431 hypermethylated DMCs were associated to 20,243 genes accounting for 37.1% of all genes present on the array. For the same cells and comparison, hypomethylation of 12.9% genes was observed. This latter could be linked, at least in part, to TET-mediated compensatory mechanism, as suggested by the significant overexpression of TET1 in GLO1-depleted cells when compared with control (Fig. S1D). Consistent DNA hypermethylation was observed in tumor xenografts generated by implantation of GLO1-depleted cells in NOD-SCID mice. Using the same array, we identified 90,441 DMCs between control and GLO1-depleted xenografts, with 79,419 (87.8%) hypermethylated DMCs and 11,022 (12.1%) hypomethylated DMCs (Fig. 1B). Differentially methylated CpGs identified using the cell line model were searched in the methylation data of the xenograft model that demonstrated a similar methylation pattern (Fig. 1C). Indeed, hypermethylated CpGs in cultured cells showed 90.1% of concordance with xenografts (Fig. S1E). Altogether these data indicate that in vitro  cell culture conditions or in vivo extracellular microenvironment did not significantly interfere with MG stress associated hypermethylation. Knowing that elevated oxidative stress can trigger DNA hypermethylation [42], we next excluded that ROS levels could have been significantly induced under both exogenous and endogenous MG stress conditions (Fig. S1F).

Upon GLO1 depletion, the hypermethylation covered intergenic, gene body and regions that regulate transcription initiation, *i.e.* gene promoters and enhancers (referred hereafter as “regulatory regions”) in MDA-MB-231 cells (Fig. 1D and Fig. S1G) and xenografts (Fig. S1H). It was however remarkable that 7% CpG probes corresponding to enhancers were hypermethylated against only 2% in promoter regions, suggesting that the effect of GLO1 depletion is more prominent on enhancer regulatory regions (Fig. 1D). Altogether, these results indicate that MG stress induced a strong DNA hypermethylation affecting a large proportion of genes including but not limited to tumor suppressors. Therefore, we next investigated if genes hypermethylated in their regulatory region could be enriched among anti-oncogenic pathways, which could suggest a potential inhibition of those pathways leading to the pro-oncogenic phenotype we have previously observed in GLO1-depleted breast cancer cells [4, 6].

### Gene set enrichment analysis (GSEA) highlights key anti-oncogenic pathways comprising MG stress-hypermethylated genes

We then performed a gene set enrichment analysis (GSEA) searching for statistical associations between the observed enhancer/promoter methylation alterations to genes under GLO1-depletion condition and those contained in a collection of pathways related to cancer development and progression [35]. We manually extracted two types of anti-oncogenic pathways from the C6 module of MSigDB (https://www.gsea-msigdb.org/gsea/msigdb/): pathways comprising TSG-activated genes (TSG-A) and those including oncogene-inhibited genes (OG-I) (Data S2). This analysis allowed the identification of 70 pathways significantly enriched in genes showing hypermethylated CpG loci (hereafter mentioned as hypermethylated pathways) with positive normalized enrichment score (NES) and FDR < 0.05 – and therefore potentially epigenetically silenced upon GLO1 knockdown. Among them, 53 belonged to OG-I and 17 represented TSG-A hypermethylated pathways. These gene sets identified as the most significantly affected by MG-associated DNA hypermethylation were notably related to kinase signaling, cell cycle regulation and cellular differentiation. The first type of gene sets, corresponding to OG-I, was related to kinase signaling and response to growth factors, notably comprising EGFR, MYC, LEF1 and PDGF enriched pathways following MG stress hypermethylation. Representative GSEA enrichment plots are shown in Fig. 1E. The second category of the main hypermethylated pathways under study, namely TSG-A, pointed to gene sets related to classic TSGs such as RB, P53 and PTEN thus, inferring to a regulatory mechanism occurring under elevated MG condition that targets TSGs primarily known to be down-regulated/inactivated through mutation in cancer. Interestingly, pathways driven by epigenetic regulators such as polycomb-repressive complex 1 (PRC1) and BMI1 were also enriched (Fig. 1E). FOXC1 gene, a target of PRC1 that is down-regulated at the mRNA level upon GLO1 depletion (Data S3), has been previously shown to inhibit migration and invasion in vitro and to reduce lung metastasis in vivo when overexpressed in MDA-MB-231 cells [43].

Importantly, hypermethylated pathways under GLO1 depletion comprised several silenced genes, as assessed based on RNASeq (Data S3) and using RT-QPCR (Fig. 1F), most of which were known as TSGs related to the metastatic process either in breast cancer or in other cancer cell types. These genes included TSGs involved in cell–cell contact and cell migratory capacity, that have been previously shown to be silenced through DNA methylation. Best examples include the epithelial to mesenchymal transition (EMT) hallmark gene E-cadherin (CDH1), whose transcriptional silencing by hypermethylation occurs at high frequency in infiltrating primary breast cancers [44]; and Ras-related protein 17 (RAB17) gene coding for a small GTPase whose expression must be reduced for tumor cells to migrate efficiently [45] (Fig. 1F). Remarkably, some metastasis-related TSGs exhibited hypermethylated CpGs in their enhancer region (eDMCs) upon MG stress. A representative example being gamma interferon-inducible lysosomal thiol reductase (GILT/IFI30) whose low expression has been associated with a higher Ki67 proliferation index and poorer survival in breast cancer [46] (Fig. 1F). The down-regulation of most of the evaluated TSGs proved to be, at least in part, dependent upon methylation as demonstrated using 5-aza-2′-deoxycytidine (5-AZA), which significantly restored gene expression in basal condition and/or under GLO1 depletion (Fig. S1I).

### DNMT3B up regulation is efficiently reversed using MG scavengers in GLO1-depleted breast cancer cells

**Fig. 2:**
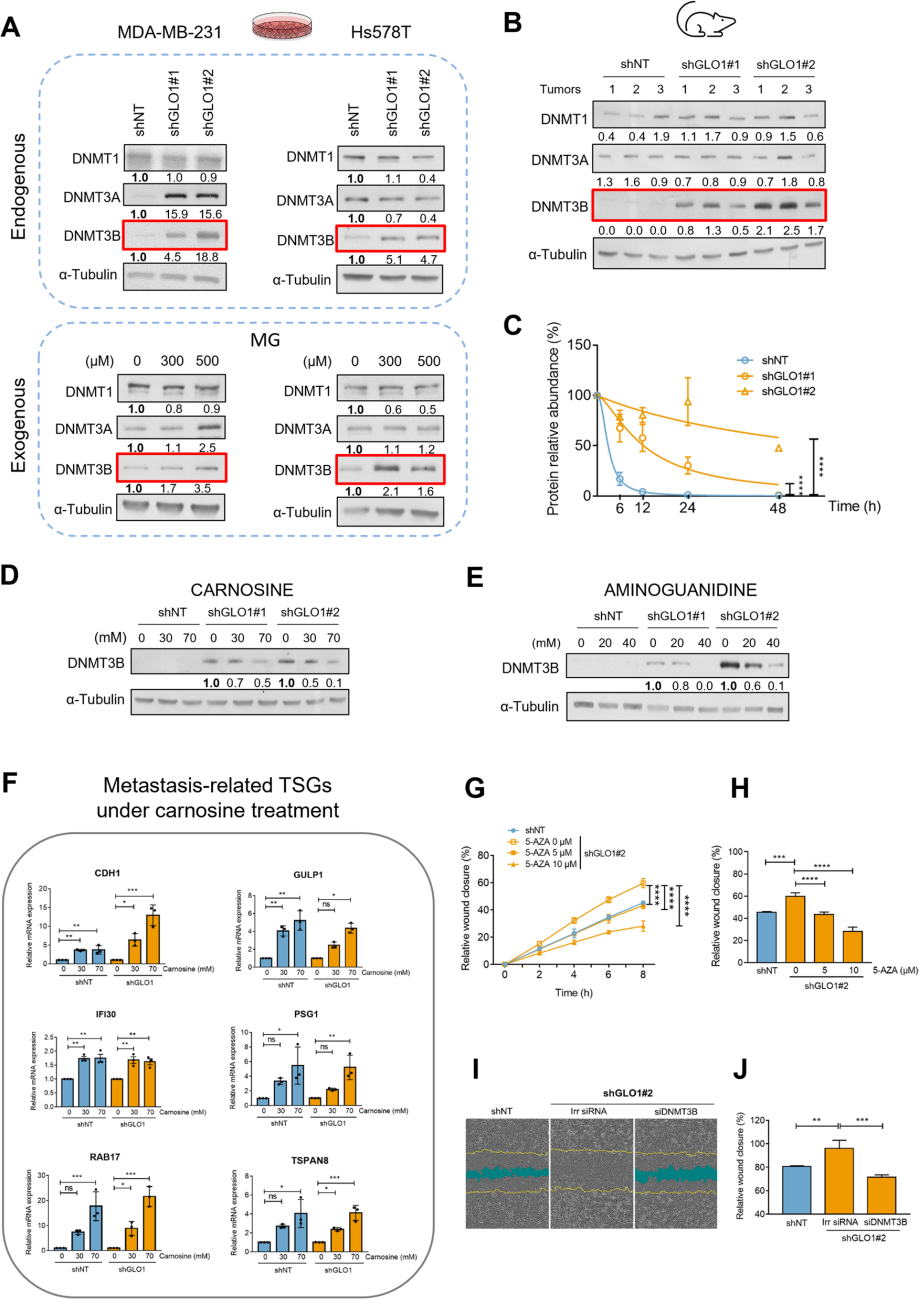
MG stress induces the overexpression of DNMT3B that is reversed using MG scavengers and that mediates enhanced migratory capacity of GLO1-depleted cells.**A** Among DNMTs, endogenous MG stress consistently increased DNMT3B protein levels across GLO1-depleted (shGLO1 #1 and #2) MDA-MB-231 and Hs578T TNBC breast cancer cells as assessed using western blot on total protein cell extracts and compared with control (shNT) cells. Additionally, exogenous MG treatment significantly up regulated DNMT3B levels in both breast cancer cell lines ^$, †^. **B** Among DNMTs, expression of DNMT3B was elevated in shGLO1 (*n* = 6) when compared with shNT mouse xenografts (*n* = 3), as assessed using western blot on total protein tumor extracts ^†^. **C** DNMT3B protein abundance, in presence of cycloheximide (10 μg/mL) at the indicated timing, in shNT MDA-MB-231 cells demonstrated shorter DNMT3B half-life contrasting with GLO1-depleted cells. Data are represented as means ± SEM of three independent experiments and were analyzed using two-way analysis of variance (ANOVA) followed by Dunnett test (**** *p* < 0.0001). Corresponding western blots are shown in Fig. S2C. **D** and** E** Carnosine (48 h) and aminoguanidine (24 h) treatments significantly reduced DNMT3B protein expression in a dose-dependent manner in GLO1-depleted MDA-MB-231 cells, respectively ^$, †^. **F** Carnosine re-induces the expression of metastasis-related TSGs under study in MDA-MB-231 cells as assessed using RT-QPCR ^§^. **G** The migratory capacity of GLO1-depleted MDA-MB-231 cells was evaluated upon 5-AZA treatment (72 h) using a scratch wound assay under Incucyte® life cell microscopy. Results are given as a percentage of relative wound closure over time for shGLO1#2 ^$, ¥^. **H** Relative wound closure at 8 h time point post scratch in shGLO1#2 cells treated with increasing doses of 5-AZA ^$, ‡^. **I** Representative pictures illustrating the wound closure at 8 h post scratch of MDA-MB-231 shGLO1#2 cells silenced (siDNMT3B) or not (Irr siRNA) for DNMT3B and compared to shNT cells. **J** Migratory capacity (8 h time point) of MDA-MB-231 shGLO1#2 cells upon DNMT3B silencing ^$, **‡**^. $ One of three experiments is shown. † Alpha-tubulin was used as a loading control. ^**‡**^ Data represent the mean values ± SD of three technical replicates and were analyzed using a one-way analysis of variance (ANOVA) followed by a Dunnett test (** *p* < 0.01, *** *p* < 0.001, **** *p* < 0.0001). § Data represent the mean values ± SEM of three independent experiments and were analyzed using a one-way analysis of variance (ANOVA) followed by a Dunnett test (* *p* < 0.05, ** *p* < 0.01, *** *p* < 0.001 and ns: not significant). ¥ Data represent the mean values ± SD of three technical replicates and were analyzed using a two-way analysis of variance (ANOVA) followed by a Dunnett test (**** *p* < 0.0001)

DNA methylation machinery essentially relies on the activity of DNMT1, DNMT3A and DNMT3B DNA methyltransferases. We next evaluated the expression of the three enzymes under both exogenous and endogenous MG stress in MDA-MB-231 cells. Under these conditions, DNMT1 basal protein level was not affected and DNMT3A expression was not consistently regulated. However, DNMT3B protein level showed a significant up regulation in GLO1-depleted MDA-MB-231 cells compared to control (Fig. 2A). DNMT3B protein up regulation was retrieved in Hs578T, another TNBC cell line stably depleted for GLO1. Acute exogenous MG challenge triggered DNMT3B induction in both TNBC cell lines (Fig. 2A), thus recapitulating GLO1 knockdown effect. Efficient GLO1 depletion at the protein level and subsequent MG adducts accumulation in Hs578T cells are shown in Figs. S2A and B, respectively. Consistent with cultured cells, was the marked increase of DNMT3B expression—but not DNMT1 and DNMT3A—in GLO1-depleted MDA-MB-231 tumor xenografts when compared with control tumors (Fig. 2B). Knowing that MG causes adduct formation on 20S proteasomal subunit proteins [47] thus likely altering the half-lives of numerous cellular proteins, we next explored the effect of GLO1 depletion on DNMT3B protein half-life using cycloheximide protein synthesis inhibitor. Interestingly, we observed that the abundance of DNMT3B over time was significantly higher in GLO1-depleted cells than in shNT cells after protein synthesis blockade (Fig. 2C). Western blots displaying DNMT3B protein stability with time are shown in Fig. S2C. Roll et al. have previously characterized a 9-gene signature associated with elevated DNMT3B expression (protein and mRNA) and DNMTs global activity, in breast cancer cell lines [48] and TNBC primary tumors [27]. We found that this DNMT3B signature effectively segregated breast cancer cells under MG stress from control cells (Fig. S2D). The silencing of 4 genes out of 9, which passed a transcript per million (TPM) count cut-off of 1 in control cells, were further validated using RT-QPCR in GLO1-depleted cells. CDH1 (shown in Fig. 1F), CST6, MUC1 and SCNN1A genes, all reported to be aberrantly hypermethylated in breast cancer, were down-regulated in both MDA-MB-231 (Fig. S2E) and Hs578T (Fig. S2F) GLO1-depleted breast cancer cells when compared with control.

Next, we reasoned that we could mimic the Warburg effect condition by switching MDA-MB-231 cells cultured in low glucose (1 g/L) to high glucose (4.5 g/L). After 24 h under high glucose condition, MG level showed a significant increase as assessed using MBo specific fluorescent probe (Fig. S2G). Along this challenge, the cells effectively showed increased DNMT3B protein level (Fig. S2H) thus recapitulating both GLO1 knockdown and exogenous MG effects. The accumulation of MG-modified HSP27, a well described MG protein adduct in cancer cells, is shown as a read out for MG stress under the same culture conditions (Fig. S2H). Then, we sought to explore whether MG stress could up regulate DNMT3B level in other human cancer cell types that we stably depleted for GLO1 expression. Interestingly, colorectal HCT116 and pancreatic MIA PaCa-2 cancer cell lines displayed significantly elevated DNMT3B protein levels upon MG stress (Fig. S2I).

We next aimed at validating further the link between GLO1 depletion and the regulation of DNMT3B cellular levels. For this purpose, we evaluated the effects of carnosine and aminoguanidine, two potent MG scavengers, on DNMT3B protein expression. Both scavengers induced a significant decrease of DNMT3B in GLO1-depleted MDA-MB-231 (Figs. 2D and E) and Hs578T cells (Figs. S3A and B). Altogether, these observations indicate that the MG stress associated to GLO1 depletion controls, at least in part, cellular availability of a de novo methylation key enzyme known to be essential for the acquisition of a hypermethylated phenotype in TNBC. Importantly, MG scavengers efficiently brought back DNMT3B expression to basal level thus pointing to their potential impact on the re-expression of methylation-silenced genes such as TSGs. Moreover, carnosine treatment effectively re-induced the expression of all the metastasis-related TSGs under study (Fig. 2F).

### The migratory advantage of GLO1-depleted breast cancer cells is lost upon inhibition of DNMT3B

Based on the data gathered so far and on our previous reports linking MG stress with the metastatic capacity of breast cancer cells [4, 6], we envisaged the possibility that MG-associated changes of the methylome may play an essential role in the launching of dominant chromatin silencing on TSGs potentially impacting on the migratory potential of breast cancer cells. Therefore, we next asked whether interfering with DNMTs activity using 5-AZA or by specifically silencing DNMT3B would affect the migratory potential of GLO1-depleted MDA-MB-231 cells. Both 5-AZA (Figs. 2G and H and Figs. S3C and D) and DNMT3B specific inhibition strategies (Figs. 2I and J and Figs. S3F and G) significantly impeded the migratory capacity of GLO1-depleted cells. Representative pictures of wound closure after silencing of DNMT3B are shown for control and GLO1-depleted cells at initial and final time points of the migration experiments under 5-AZA (Fig. S3E) and siDNMT3B conditions (Fig. 2I and S3G). Efficient decrease of DNMT3B protein level was verified under the same experimental conditions (Figs. S3H and I). These results show that DNMT3B is implicated in the acquisition of an enhanced migratory phenotype in MG-stressed TNBC cells. At this stage of the study, we demonstrated that MG stress is an upstream epigenetic regulator process that impacts on the hypermethylation of metastasis-related TSGs and the pro-migratory capacity of breast cancer cells.

### Integrative analysis of DNA methylation and gene expression data identifies a specific MG stress signature

**Fig. 3:**
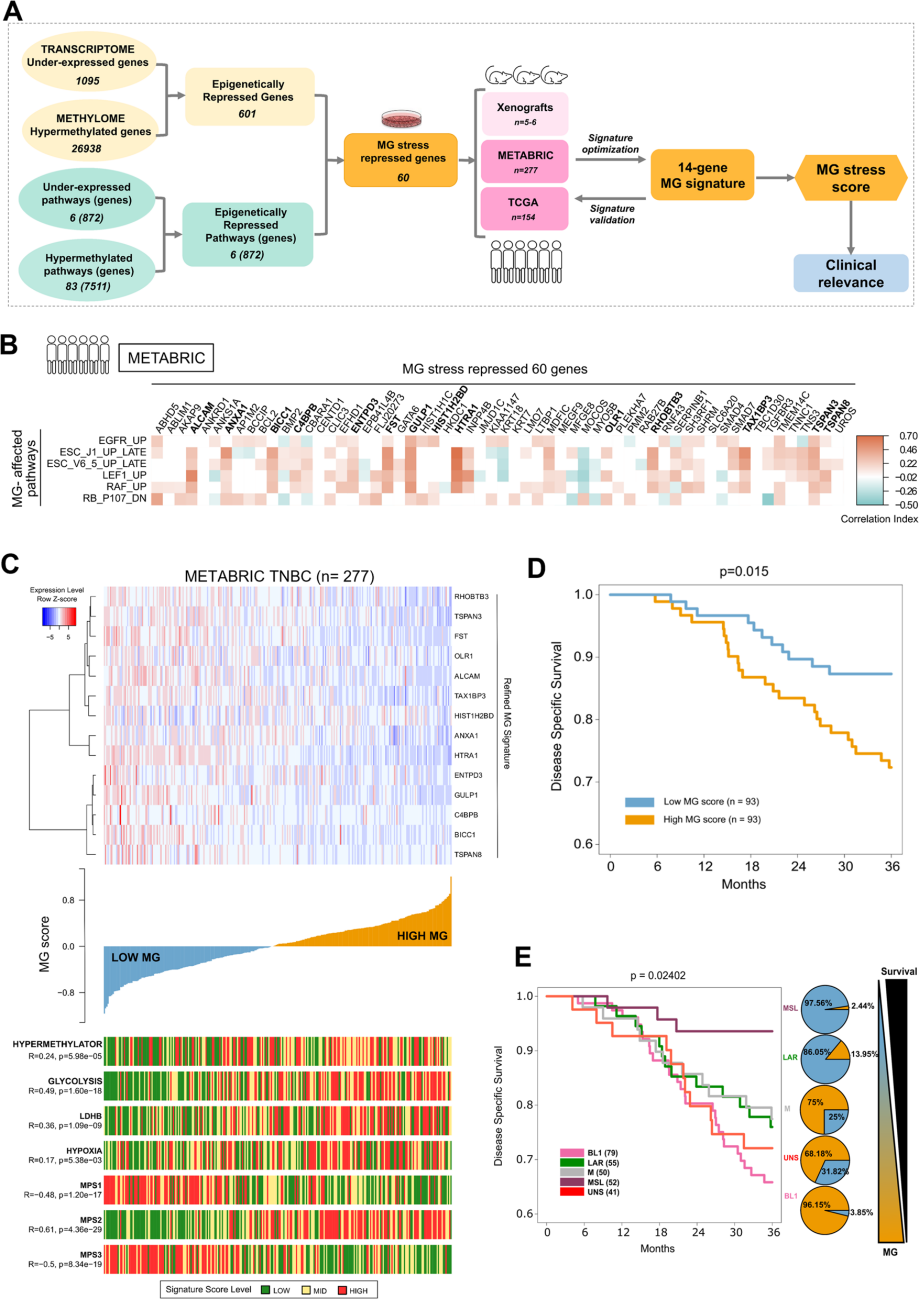
Integrative analysis of DNA methylation and gene expression data identifies a 14-gene signature of MG stress with clinical relevance.**A** Workflow showing the integration of differential gene expression, methylation and their corresponding GSEA pathway enrichment under MG stress condition. This integration led to the selection of 60 potential candidates that represented genes repressed under MG stress. These 60 genes were validated in xenografts and refined further using pathway correlation approach (detailed in Material and Methods) using TNBC patient data from METABRIC cohort. This resulted in a final 14-gene based signature of MG stress. Next, MG score was derived from this MG signature and was evaluated for its clinical relevance in TNBC patients. The numbers in the yellow boxes correspond to the genes resulting from the indicated analysis. The numbers in the green boxes represent GSEA pro-oncogenic pathways with their respective total number of genes specified between brackets. **B** Signature optimization data are represented in a heatmap showing correlation between the six MG stress-affected pathways (rows) and 60 integration genes (columns) using METABRIC TNBC patient cohort. Genes with statistically significant correlation greater than 0.25 with at least one of the pathways were selected for composing the final MG signature. The outcome of this optimization step is 14 genes highlighted in bold in the columns and composing MG signature (detailed in Material and Methods). Positive and negative correlations are shown in red and cyan colors, respectively. Insignificant correlation values (*p*-value > 0.05 or correlation coefficient = 0) are shown as white boxes. **C** Top panel represents a heatmap of the 14-gene MG signature in METABRIC TNBC patients (*n* = 277). Middle panel shows the waterfall plot representing TNBCs distribution from low to high MG score (Y-axis). Bottom panel represents signature status of hypermethylator phenotype [27], metabolic glycolysis and hypoxia signatures based on Reactome gene lists, LDHB gene expression, and metabolic-pathway-based subtypes (MPS1, MPS2, MPS3) [53]. All these signature scores are represented as low (green), mid (yellow) and high (red) level and their respective Spearman correlation (R) with p-values is given when compared to MG signature score. **D** Kaplan–Meier curves showing significant differences (*p* = 0.015) in disease specific survival between low (*n* = 93) and high (*n* = 93) MG score TNBC tumors from METABRIC cohort. **E** Kaplan–Meier curves showing significant differences of disease specific survival across Lehmann subtypes using METABRIC TNBC cohort with respective MG score status highlighted in the pie plots shown beside. As MG score increases the survival probability decreases, with patients bearing BL1 and UNS tumor subtypes being among the worst survivors. Respective number of patients for each TNBC subtype are mentioned in parentheses

In order to gain more insight into the genes epigenetically regulated upon GLO1 depletion, we next undertook an integrative analysis using methylome and transcriptome data of shGLO1#2 cells, which displayed the most efficient reduction of GLO1 and thereafter mentioned as shGLO1 (Fig. S1A). First, we implemented Kallisto-Sleuth pipeline (see Materials and Methods) to analyze the transcriptomic data generated from shGLO1 and shNT MDA-MB-231 cells. We found 2018 differentially expressed genes (DEGs), among which 1095 genes were down-regulated and 923 genes were up-regulated. Secondly, we identified 26,938 differentially hypermethylated and 9553 hypomethylated genes. We integrated the aforementioned transcriptomic and methylome results (as described in Materials and Methods) to generate two lists of genes showing an inverse relation between their methylation level (promoter and/or enhancer regions) and their expression level under MG stress: hypermethylated/low expression (Data S3) and hypomethylated/high expression (Data S4). This first step of integration resulted in 601 hypermethylated and down-expressed genes that represented epigenetically repressed genes in GLO1-depleted cells (see diagram in Figs. 3A and S4A). Interestingly, the ToppFun gene ontology analysis run on epigenetically repressed genes recapitulated most of the metastasis-related biological processes that we have previously highlighted based on MG stress transcriptome only [6] (Fig. S4B). Thus suggesting that these oncogenic processes are likely associated with the tumor suppressor genes identified as epigenetically repressed in the present study.

In the second step of integration (Fig. 3A), GSEA analysis of methylation data, based on the anti-oncogenic gene sets described above (Data S2), allowed the identification of 83 significant (FDR < 0.05) pathways with positive normalized enrichment score (NES) for methylation (hypermethylated pathways), that are affected upon induction of MG stress. GSEA analysis of shGLO1 transcriptomic data revealed six significant pathways with negative NES (FDR < 0.05) for gene expression (underexpressed pathways) (Fig. 3A and Data S2). Intersection of the hypermethylated and underexpressed pathways resulted in six common anti-oncogenic pathways expected to be epigenetically repressed under MG stress. The final intersection between the 872 genes composing these six epigenetically repressed pathways and the 601 hypermethylated genes resulted in 60 genes that generated the ‘60-gene MG signature’ (Fig. 3A, Data S7). This signature summarizes MG pro-cancer effects at the gene and pathway levels that resulted from either OG-I or TSG-A control. Importantly, when verified, majority of the genes composing the 60-gene MG signature derived from cell lines consistently showed higher methylation levels in GLO1-depleted xenografts compared to control (Fig. S4C). Prompted by this observation, we performed RT-QPCR on GLO1-depleted tumor xenografts and validated the loss of expression of several genes composing the six main deregulated anti-oncogenic pathways and thus belonging to the 60-gene MG signature (Fig. S4D). The significant loss of cytokeratin 18 (KRT18), a well described luminal breast cancer marker, is worth mentioning, as the sole knockdown of KRT18 has been recently shown to induce EMT and stemness features in non-metastatic MCF-7 breast cancer cells [49]. Even more supportive of its TSG function, the forced expression of KRT18 in MDA-MB-231 cells has been reported to abolish their capacity to form tumors and metastasize in vivo [50]. Another significantly repressed TSG under MG stress is phosphatidylinositol phosphatase (INPP4B) gene, the loss of which represents one of the two best biomarkers (with nestin gain) for the identification of basal-like subtype [51]. Interestingly, the MG signature notably comprised follistatin (FST), a member of the transforming growth factor-β ligands, previously reported as a metastasis suppressor in breast cancer [52].

In sum, this upper level of integration selected for six epigenetically repressed pathways included three TSG-A gene sets, the RB pathway and two pathways related to late stages of embryonic stem cell (ESC) differentiation (ESC J1 and ESC V6.5) that are potential suppressors of cancer stem cell phenotype; and three OG-I gene sets, the EGFR and RAF1 main drivers of stem cell and lymphoid enhancer-binding factor 1 (LEF1), as detailed in Data S2. The integrative analysis of DNA methylome and transcriptome highlighted cell cycle control, cell differentiation and kinase signaling, all processes that are consistent with a pro-oncogenic role for MG stress-mediated hypermethylation in breast cancer.

### Optimization of MG stress signature based on epigenetically repressed pathways in TNBC patients

In order to assess MG stress in patients, we aimed at optimizing the MG signature in a TNBC cohort. For this purpose, we used the expression data from the Molecular Taxonomy of Breast Cancer International Consortium (METABRIC) cohort comprising 277 TNBC primary tumors to identify, from the 60-gene signature, the genes that best reflected MG affected pathways in cultured TNBC cells. For this identification, only the genes presenting a significant positive correlation (Pearson’s correlation coefficient R > 0.25, *p*-value < 0.05) with their respective pathway were considered as candidate genes for the refined MG signature (Fig. 3B). At this stage of the study, integrated methylation and expression data analysis along with pathway enrichment assessment resulted in a comprehensive MG signature of 14 known or novel TSGs, whose expression was epigenetically repressed in breast cancer. This refined 14-gene MG signature was then taken forward for clinical association studies and analysis.

### MG signature score correlates with key methylation, metabolic and clinical features in TNBC tumors

Next, we tested the 14-gene signature for its clinical relevance using gene expression and patient clinical data from METABRIC cohort (Fig. 3C, top panel and Data S8). First, an MG score was calculated for each patient using its expression data, the 14-gene MG signature and the function ‘signature_score’, as described in Materials and Methods. Then, breast cancer patients were ordered from low to high MG score groups (Fig. 3C, middle panel). For validating the relation between the 14-gene MG signature and methylation status in TNBC tumors, we correlated MG score with the score calculated for the breast cancer hypermethylator phenotype signature [27], revealing an expected positive correlation (R = 0.24, *p*-value = 5.98e^−05^) (Fig. 3C, bottom panel). MG score also proved to be significantly correlated with pathways regulating glycolysis (R = 0.49, *p*-value = 1.60e^−18^) and hypoxia (R = 0.17, *p*-value = 5.38e^−03^) (Fig. 3C, bottom panel, signatures from Reactome gene lists).

We next referred to a study recently published by Gong and collaborators [53] who proposed the clustering of TNBC patients, of the Fudan University Shanghai Cancer Center (FUSCC) cohort, into three metabolic-pathway-based subtypes (MPS1, MPS2 and MPS3) presenting characteristic metabolic vulnerabilities that can be therapeutically targeted. We took advantage of MPS classification to understand how MG signature aligns and correlates with each metabolic subtype and may contribute to metabolism-based TNBC subtyping. We found that MG score was strongly correlated with MPS2 (R = 0.61, *p*-value = 4.36e^−29^), the glycolytic TNBC subtype with up regulated carbohydrate and nucleotide metabolism (Fig. 3C, bottom panel). Interestingly, we observed that MG score showed a strong negative correlation with MPS1 signature (R = -0.48, *p*-value = 1.20e^−17^) representing lipogenic TNBCs and MPS3 (R = -0.50, *p*-value = 8.34e^−19^) corresponding to TNBCs displaying a mixed metabolic subtype [53]. MG stress was positively correlated with lactate dehydrogenase B (LDHB), a biomarker of the glycolytic phenotype that is overexpressed in TNBC tumors with poor clinical outcome [54] (Fig. 3C, bottom panel). It is noteworthy that MPS2 TNBCs showed the worse relapse free survival when compared with other MPS subtypes and were sensitive to anti-LDHB therapy [53]. Altogether our results sustain the strong correlation existing between MG stress-related epigenetic changes and TNBC glycolytic phenotype and let us propose that MG signature and MPS2 metabolomic phenotypes converge to identify a subset of TNBC patients with worst survival. To validate further the value of our 14-gene signature for the classification of TNBC tumors, we next analyzed the expression data of TNBCs (*n* = 154) from The Cancer Genome Atlas (TCGA) cohort. In line with the correlations observed using METABRIC cohort, this second collection highlighted a subgroup of breast tumors with high MG scores that also presented high methylation and glycolysis calculated scores (Fig. S5A and Data S9). Collectively, these results externally validate MG stress occurrence in human breast tumors across different cohorts.

TNBCs are generally poorly differentiated tumors with unfavorable outcome. Our analysis indicated that MG score was elevated from most to least histologically differentiated TNBC tumors and discriminated moderately (grade 2) from poorly (grade 3) differentiated tumors (*p*-value = 2.6e^−06^) (Fig. S5B). This is consistent with fast growing and more likely invasive histologic grade 3 tumors undergoing a marked glycolytic switch [55] and prone to acquire an MG stress gene signature.

Importantly, global Kaplan Meier analysis revealed significant segregation (*p*-value = 0.015) between high MG and low MG scored METABRIC patients in terms of disease specific survival (DSS, Fig. 3D) and overall survival (OS, Fig. S5C), with patients bearing high MG score tumors presenting the poorest survival.

Based on gene expression profiling analyses, Lehman and collaborators [56, 57] have delineated a widely recognized classification for TNBCs. We used the distribution of Lehmann subtypes in the METABRIC TNBC dataset and further correlated this with MG score level. High MG score occurred in a majority of basal-like 1 (BL1) subtype followed by unstable (UNS) and mesenchymal (M) subtypes, indicating that MG stress allows for the specific characterization of TNBC tumors across different Lehman subgroups. Even more remarkable is that BL1 and UNS high MG-stress subtypes represented TNBC patients with shorter DSS (Fig. 3E) and OS (Fig. S5D) when compared with the other Lehmann subtypes.

Collectively, our findings contribute to position MG stress in TNBC as a novel targetable biomarker impacting on DNA methylation, which could effectively sign the glycolytic phenotype of a subgroup of poor prognosis TNBC tumors that may be sensitive to MG scavenger-based therapy.

## Discussion

It is admitted that abnormal patterns of DNA methylation localized gains have profound impact on gene expression during malignant tumor development and metastatic progression in cancer patients. As an emerging hallmark of cancer cells, the role of metabolic reprogramming is becoming increasingly important. Original connections recognized between metabolism and epigenetic regulation were mainly based on metabolism issued substrates and co-factors that, directly or secondarily, favor or limit the activity of chromatin-modifying enzymes [58]. Glycolysis products such as pyruvate [59] and lactate [60] have been shown to decrease the expression of histone deacetylases (HDACs). Most of published studies on MG-mediated post-translational modifications have focused on hydroimidazolone adducts on arginine residues, likely due to their abundance and/or availability of specific polyclonal antibodies. Coukos and collaborators [61], using an elegant chemoproteomic approach, have generated the first proteome-wide landscape of MG modification of cysteine residues, with key cysteines occurring in the catalytic site of major metabolic enzymes such as acetyl-coenzyme A acetyltransferase (ACAT1). Metabolites playing a key role in DNA methylation such as homocysteine, a methyl cycle intermediate comprising a thiol group, could be potentially targeted byMG. No doubt that the continued characterization of MG protein targets and their functions will contribute to a better positioning of MG oncometabolite in cancer progression and metastasis development.

Our data, demonstrating that MG stress acts as a regulator of DNA methylation, are a step toward enlightening the multifaceted cross-talk existing between energy metabolism and epigenetic silencing in cancer. In GLO1-depleted cells, we observed minor hypomethylation and major DNA hypermethylation. The hypomethylation can potentially be explained by a compensatory mechanism, as we observed TET1 overexpression in GLO1-depleted cells. Also, in rapidly replicating cells (feature of cancer cells) it is known that the maintenance of DNA methylation during DNA replication could be compromised thus allowing “passive demethylation” [62, 63]. Concerning the hypermethylation, we observed that the induction of MG stress caused an elevated DNMT3B protein level resulting, at least in part, from a prolonged cellular half-life in glycolytic and metastatic breast cancer cells. Importantly, we have shown that these cells lose their migratory capacity upon DNMT3B knockdown thus illustrating the link between MG stress pro-cancer phenotype and altered DNA methylation machinery.

Remarkably, most of the cancer gene signatures reported to date are based on oncogene-driven regulation of gene expression as an outcome predictor in breast cancer [64, 65]. In comparison, TSGs have been poorly appreciated as potential indicators of prognosis and/or response to therapy. In this study, aberrant DNA methylation under MG stress led us to characterize a 14-gene signature consisting of hypermethylated and down-regulated genes that is significantly correlated with poor survival in TNBC patients. Hypermethylation affected promoter and/or enhancer regions of specific TSGs known primarily for their role in regulating the metastatic potential of cancer cells such as: ENTPD3, an inhibitor of EMT and metastasis in breast cancer [66], and ALCAM cell adhesion molecule which reduced expression has been associated with poor prognosis in a large TMA-based immunohistochemical analysis of breast cancer [67].

There are several implications for this newly identified role of MG stress in DNA methylation. First, it mechanistically sheds light on both known and novel metastasis-related TSGs that are epigenetically inactivated in glycolytic TNBC cells. More specifically, it brings a new approach for explaining the loss of TSG pressure in breast cancer that could result from metabolic alterations rather than mutations. We showed that MG stress exerted its regulatory effect on DNMT3B levels in breast, colon and pancreatic cancer cells. Future studies will prove useful in expanding our original findings to the numerous other cancer types that are characterized by oncogene-driven glycolytic reprogramming, as observed in most of melanoma tumors for example. Another potentially important implication of linking MG stress and epigenetic deregulation concerns our understanding of the increased risk of cancer in diabetic patients [68]. MG has been directly related to the development of diabetes [69] and its complications [70]. Our results suggest that the alterations exerted by MG stress on the epigenome may contribute to the growing evidence supporting the role of epigenetic regulation not only as a trigger but also as a response to obesity and type 2 diabetes [71].

Several MG-repressed TSGs in this study are typically known to be less expressed in basal than in luminal breast cancer cells. Intriguingly, MG likely imposed an “exacerbated” epigenetic repression on genes already expressed at low levels in basal breast cancer cells. The down-regulation of ESR1, CDH1 and FST genes under MG stress is the best example of this phenomenon. We propose that MG stress could represent an upstream mechanism responsible, at least in part, for the acquisition of a basal-like phenotype. Additional studies using luminal breast cancer MG stress models are needed to explore further this inspiring observation. Complementary to the seminal Knudson’s two-hit model that initially allowed the identification of TSGs [72], it is important to also consider the expression levels of TSGs and not only assess mutations within these genes. Although difficult to measure and normalize, subtle changes in TSG expression levels could influence cancer progression and metastasis, as it is now recognized for oncogenes (*i.e.* Myc amplification or KRAS hyperactivation).

Alterations in DNA methylation patterns have been tracked in order to target metastatic dissemination and chemoresistance: the two most critical challenges for TNBC patients. We have previously reported that MG scavengers inhibit the migratory capacity of breast cancer cells [6] and repress their metastatic potential in vivo [4]. In this study, we show that MG scavengers are novel powerful down-regulators of DNMT3B provoking the re-expression of TSGs implicated in key processes such as differentiation, cell cycle control and apoptosis; all therapeutically desirable effects. Although further preclinical confirmation is required, MG scavengers such as carnosine (an endogenously produced dipeptide) and aminoguanidine (used to reduce diabetes complications) represent a novel and promising non toxic treatment option for TNBC tumors with high MG score.

Considering the subtle crosstalk existing between histone modification and DNA methylation, it is well established that their mutual influence contributes to genomic instability and cancer progression. For example, de novo DNA methylation in cancer is possibly targeted to some genes marked with histone H3 lysine 27 methylation Interestingly, the other way around, DNA methylation might serve as a template for some histone modifications after DNA replication [73]. Based on this, we do not exclude that MG-mediated glycation of histones may influence DNA methylation either directly with glycated-histones defining a novel histone code, yet to be deciphered, that may favor the recruitment of specific writer and/or eraser proteins; or indirectly as it has been shown that some histones see their canonical post-translational modifications (*i.e.* acetylation) hampered by MG-mediated glycation [20].

Consequently, it is expected that the complex molecular mechanisms needed to trigger the re-activation of epigenetically silenced TSGs requires the concomitant use of DNA hypomethylating agents and inhibitors of histone deacetylases such as trichostatin A or LAQ824. Previous use of such combined treatments resulted in a synergistic reversal of TSG silencing in MDA-MB-231 cells [74]. Taking into account that MG induces DNA hypermethylation and the glycation of core histones in breast cancer cells, the use of MG scavengers appears to be an attractive, but yet to be demonstrated, “one stone two birds” option in view of the recognized roles of epigenetic regulation in tumor initiation, progression and resistance following therapeutic intervention.

## Conclusion

Our study complements the recent positioning of MG stress as a key regulatory mechanism of histone epigenome and brings a new dimension to the use of DNMTs inhibitors for the personalized treatment of patients bearing specific subtypes of TNBC.

## Supplementary Information

**Additional file 1.**

## Data Availability

The Infinium DNA methylation data related to this study are publicly available in the NCBI’s GEO database (GEO ID: GSE185237).

## Abbreviations

GLO1: Glyoxalase 1
MG: Methylglyoxal
TNBC: Triple negative breast cancer
TSGs: Tumor suppressor genes
