# Supplementary material for: Methylglyoxal: a novel upstream regulator of DNA methylation
Source: J Exp Clin Cancer Res. 2023 Mar 31;42:78. doi: 10.1186/s13046-023-02637-w (PMC10064647; doi:10.1186/s13046-023-02637-w)
Supplement: Supplementary file 1 — Additional file 1. [file 13046_2023_2637_MOESM1_ESM.zip › Additional File 1.docx]

**Fig. S1. A.** Efficient GLO1 silencing in MDA-MB-231 cells (shGLO1#1 and shGLO1#2) compared to control (shNT) was assessed using western blot. One of three experiments is shown. Alpha-tubulin/Beta-actin was used as a loading control. **B.** Principal component analysis (PCA) plot showcasing the distinct clustering of shGLO1 (n=6) and shNT (n=3) samples. **C.** Scatter plot of differentially methylated CpG sites, where X-axis and Y-axis represent β-values from 850K Infinium array and pyrosequencing experiments, respectively. P-value and estimate values from Pearson correlation results are indicated at the top. **D.** Ten eleven translocation (TET) enzymes mRNA levels were evaluated using QRT-PCR in GLO1-depleted MDA-MB-231 cells. Data represent the mean ± SEM of three biological replicates and were analyzed using unpaired t-test (** p < 0.01, *** p < 0.001 and ns: not significant). **E.** Venn diagram representing the proportion of concordant hypermethylated CpGs between cultured MDA-MB-231 cells and MDA-MB-231 mouse xenografts. **F.** Reactive oxygen species (ROS) levels were assessed in MDA-MB-231 cells by flow cytometry using DCFDA probe upon MG treatment (upper panel) at the indicated concentrations when compared to untreated cells (CTRL), and in GLO1-depleted cells (lower panel). Data are represented as mean ± SEM of 3 biological replicates and were analyzed using unpaired t-test (*** p < 0.001 and ns: not significant). **G.** Proportion of hypo- and hypermethylated CpGs distributed across intergenic and gene body genomic regions. **H.** Proportion of hypo- and hypermethylated DMCs distributed across the different genomic regions in MDA-MB-231 mouse xenografts. Mixed regions correspond to Infinium array probes referring to either promoter or enhancer, according to the considered cell line. **I.** 5-AZA 72h-treatment re-induced mRNA expression of all, with the exception of GULP1 and TSPAN8, the metastasis-related TSGs under study as assessed using RT-QPCR. Gene expression in treated shNT and shGLO1 cells is shown as a fold-change relative to the non-treated cells arbitrarily set to 1. Data represent the mean ± SEM of three independent experiments and were analyzed using one-way analysis of variance (ANOVA) followed by Dunnett test (* p < 0.05, ** p < 0.01, *** p < 0.001 and ns: not significant).

**Figure S2. A.** Efficient GLO1 silencing in Hs578T cells (shGLO1#1 and shGLO1#2) compared to

control (shNT) as assessed using western blot ^$, †^. **B.** Accumulation of argpyrimidine (ArgPyr) MG protein adducts in Hs578T shNT, shGLO1#1 and shGLO1#2 cells was detected using western blot ^$, †^. **C.** DNMT3B detection, in presence of cycloheximide (10μg/mL) treatment at the indicated timing, in control (shNT) MDA-MB-231 cells demonstrated shorter DNMT3B half-life contrasting with GLO1-depleted cells (shGLO1#1 and #2). Western blots densitometric values are shown graphically in Figure 2C as percentages of time 0h value for each condition at different time points after cycloheximide treatment ^$, †^. **D.** Heatmap based on DNMT3B overexpression signature (27) as observed in RNAseq data of GLO1-depleted (n=6) compared to shNT (n=3) cells. Orange: low expression, blue: high expression. **E. and F.** Validation of representative genes of the Roll et al. DNMT3B overexpression signature (27) using RT-QPCR in GLO1-depleted cells (shGLO1#1 and shGLO1#2) compared with control (shNT) in MDA-MB-231 (see also CDH1 data in Figure 1F) and Hs578T cell lines, respectively. mRNA levels in shGLO1 cells are represented as fold-change relative to shNT level arbitrarily set to 1. Data represent the mean ± SEM of three independent experiments and were analyzed using one-way analysis of variance (ANOVA) followed by Dunnett test (* p < 0.05, ** p < 0.01, *** p < 0.001 and **** p < 0.0001 and ns: not significant). **G.** MG levels were assessed by flow cytometry after adding MBo fluorescent probe to MDA-MB-231 cells cultured in low glucose (LG, 1 g/L) or high glucose (HG, 4.5 g/L). Data represent the mean ± SEM of three independent experiments and were analyzed using unpaired t-test (* p < 0.05). **H.** Western blot assessment highlighting elevated DNMT3B levels in high glucose (HG) when compared to low glucose (LG) setting in MDA-MB-231 cells. MG-HSP27 represents MG-modified HSP27 as a readout of MG stress under the same conditions of culture and at the indicated timing ^$, †^. **I.** Endogenous MG stress consistently increased DNMT3B protein levels across GLO1-depleted (shGLO1) colorectal HCT 116 and pancreatic MIA PaCa-2 cancer cells as assessed using western blot on total protein cell extracts and compared with the corresponding control (shNT) cells ^$, †^. $ One of three experiments is shown. † Alpha-tubulin/Beta-actin was used as loading control.

**Figure S3.** **A. and B.** Carnosine (48h) and aminoguanidine (24h) treatments significantly reversed and reduced DNMT3B protein expression in a dose-dependent manner in Hs578T cells, respectively ^$, †^. **C.** The migratory capacity of MDA-MB-231 GLO1-depleted and shNT cells was evaluated upon 5-AZA treatment (72h) under Incucyte^®^ life cell microscopy. Results are given as a percentage of relative wound closure over time for shGLO1#1 ^$, ¥^. **D.** Relative wound closure at 8h time point post scratch in shGLO1#1 cells treated with increasing doses of 5-AZA ^$, ‡^. **E.** Representative pictures illustrating the wound closure at 8h post scratch of MDA-MB-231 shGLO1#2 cells were evaluated upon 5-AZA treatment (72h) in a scratch wound assay (Incucyte^®^) and compared to control cells (shNT). **F.** Representative pictures illustrating the wound closure at 8h post scratch of MDA-MB-231 shGLO1#1 cells silenced (siDNMT3B) or not (Irr siRNA) for DNMT3B and compared to control cells (shNT). **G.** Migratory capacity (8h time point) of MDA-MB-231 shGLO1#1 cells upon DNMT3B silencing compared with shNT cells ^$, ‡^. **H.** 5-AZA treatment decreased DNMT3B expression after 72h in the indicated MDA-MB-231 breast cancer cells ^$, †^. **I.** Effective silencing of DNMT3B protein expression is shown after transfection with DNMT3B specific siRNA when compared with irrelevant siRNA (Irr siRNA) used as control in MDA-MB-231 shGLO1#1 and shGLO1#2 cells ^$, †^. $ One of three experiments is shown. † Alpha-tubulin was used as loading control. ^‡^ Data represent the mean values ± SD of three technical replicates and were analyzed using a one-way analysis of variance (ANOVA) followed by a Dunnett test (** p < 0.01, *** p < 0.001 and **** p < 0.0001). ¥ Data represent the mean values ± SD of three technical replicates and were analyzed using a two-way analysis of variance (ANOVA) followed by a Dunnett test (**** p < 0.0001).

**Figure S4**. **A.** Volcano plot summarizing integration of gene expression and methylation data of GLO1-depleted breast cancer cells. The 601 hypermethylated and down expressed genes are highlighted in the left box and the right box shows 135 hypomethylated and high expressed genes. **B.** ToppFun gene ontology results comparing metastasis related enriched biological processes identified between differentially expressed genes from Nokin et. al. (6) and epigenetically repressed genes from the current study. **C.** Heatmap representing the methylation levels and unsupervised clustering of the 60-genes (rows) repressed under MG stress in MDA-MB-231 cells were validated in shNT (n=3) and shGLO1 mouse xenografts (n=3) (columns) methylation data. When several CpGs corresponded to one gene, the one showing the highest absolute Δβ value in cultured cells was selected. Color key scale represents methylation β-values (blue: low methylation and orange: high methylation). **D.** MDA-MB-231 GLO1-depleted xenografts (shGLO1) show a significant decrease of 8 selected genes from the 60 MG-repressed genes compared to shNT tumors. Each point of the graph represents a mouse (n=5-6). Data represent the mean ± SEM and were analyzed using unpaired t-test (*** p < 0.001 and **** p < 0.0001).

**Figure S5. A.** Top panel represents a heatmap of the 14-gene MG signature in TCGA TNBC patients (n=154). Middle panel shows the waterfall plot representing TNBCs distribution from low to high MG score (Y-axis). Bottom panel represents signature status of hypermethylator phenotype (27), metabolic glycolysis and hypoxia signatures based on Reactome gene lists, LDHB gene expression, and metabolic-pathway-based subtypes (MPS1, MPS2, MPS3) (53). All these signature scores are represented as high (red), mid (yellow) and low (green) level and their respective Spearman correlation (R) with p-values is given when compared to MG signature score. **B.** Boxplot representing the MG score levels across the tumor grade of TNBC patients. **C.** Kaplan-Meier analysis showing significant overall survival probability (p=0.007) between low (n=93) and high (n=93) MG score TNBC tumors from METABRIC cohort. **D.** Kaplan-Meier analysis showing significant differences in terms of overall survival probability (p=0.021) across TNBC Lehmann subtypes from METABRIC cohort with respective MG score status highlighted in the pie plots shown beside. As MG score increases, the survival probability decreases with patients bearing BL1 and UNS tumor subtypes being among the worst survivors. Respective number of patients for each TNBC subtype is mentioned in parentheses.

**Table S1.** PCR amplification and sequencing primers used for bisulfite pyrosequencing experiments.

| **Target** | **Name** | **Sequence** | **Type** | **Tm** (°C) |
| --- | --- | --- | --- | --- |
| MAPK8IP2 | MAPK8IP2 F | AGAGTAGATTTATAGGGTTGTGTTTAGG | Forward | 59 |
| MAPK8IP2 | MAPK8IP2 R | [Btn]ACCTCACCCAACAAATCCTTAA | Reverse | 59 |
| MAPK8IP2 | MAPK8IP2 S | GTGGATGATTTTGTGTTG | Sequencing |  |
| SOX14 | SOX14 F | [Btn]AAAGGTTAGGAGAGAAGTAGATTAGG | Forward | 57 |
| SOX14 | SOX14 R | CACCACCTAAAATTTAACCCCAAACTACC | Reverse | 57 |
| SOX14 | SOX14 S | CACATTTACCTAAAAAAACTATAC | Sequencing |  |
| AKAP12 | AKAP12 F | [Btn]GGGGGATTAGTGGAGAATGGAT | Forward | 61 |
| AKAP12 | AKAP12 R | AACCAACTACACCCCAAACTTTCTCT | Reverse | 61 |
| AKAP12 | AKAP12 S | ACCCCAAACTTTCTCTA | Sequencing |  |
| SLC6A3 | SLC6A3 F | AGAGTTGGGAGGAGGATGGATA | Forward | 58 |
| SLC6A3 | SLC6A3 R | [Btn]AAAACACCCTACTACTAAATCCAAATAACA | Reverse | 58 |
| SLC6A3 | SLC6A3 S | TGGGTTAGTAGGGTAA | Sequencing |  |
| SLC6A3 | SLC6A3 nested F | AAGTGTTTGTATAAGTTAT | First round nested PCR | 48 |
| SLC6A3 | SLC6A3 nested R | CCTATTAAAACCTCCACCTAC | First round nested PCR | 48 |
| DUOXA2 | DUOXA2 F | [Btn]TTTAGATTAGTTTTTGGGTTGGGATAT | Forward | 57 |
| DUOXA2 | DUOXA R | ACAAACCAACAAACTAAAACTACCT | Reverse | 57 |
| DUOXA2 | DUOXA2 S | CTATAACCCTTACTTTCCTAATTCT | Sequencing |  |
| DUOXA2 | DUOXA2 nested F | AGTGTGAGTATTTGGATTTAG | First round nested PCR forward | 50 |
| DUOXA2 | DUOXA2 nested R | CCTACACTATAAAAACACC | First round nested PCR Reverse | 50 |
| DOC2B | DOC2B M F | TGTGTAGAGATAAGTGGTGGAAGTAT | Forward | 52 |
| DOC2B | DOC2B R | [Btn]ACCTATACCCTAAATACCCTTAATC | Reverse | 52 |
| DOC2B | DOC2B S | AGATAAGTGGTGGAAGTATT | Sequencing |  |
| DOC2B | DOC2B nested F | GTAGGTGTGTAAGTGTATATATG | First round nested PCR forward | 48 |
| DOC2B | DOC2B nested R | TCAAACTATACTCACTAACC | First round nested PCR Reverse | 48 |
| KMT2B | KMT2B N F | GGTTTGGGAGAGGAGGTAGT | Forward | 59 |
| KMT2B | KMT2B R | [Btn]CCCTCCACCACTCTACCTAAA | Reverse | 59 |
| KMT2B | KMT2B S | GTGGGGATTGTATGTTTA | Sequencing |  |
| RASGRP1 | RASGRP1 F | GGGGAGAGGAGTGTAGTTTT | Forward | 52 |
| RASGRP1 | RASGRP1 R | [Btn]CCACCTAATAACCCCTCC | Reverse | 52 |
| RASGRP1 | RASGRP1 S | GGGTAATAGAGGGGTGTT | Sequencing |  |
| RASGRP1 | RASGRP1 nested F | GTGAATGGAGTTGTGAAGTG | First round nested PCR forward | 48 |
| RASGRP1 | RASGRP1 nested F | CCTATATCCTTTCTTAAA | First round nested PCR Reverse | 48 |
| OLIG2 | OLIGO2 F | AGTAGAGTATTAAGATAGTGGGGATTT | Forward | 55 |
| OLIG2 | OLIGO2 R | [Btn]CTATTCCCATCTCACTAATTTCATCAAC | Reverse | 55 |
| OLIG2 | OLIGO2 S | GGGTATAGTTTATATTTGGGG | Sequencing |  |
| TDRKH | TDRKH V F | [Btn]AGGAGGTTGAGGGGTTTT | Forward | 56 |
| TDRKH | TDRKH R | AATCCTCCAAAACTAAACTCTTCCCTATC | Reverse | 56 |
| TDRKH | TDRKH S | AACCCAATCAAACTAAATAAACTTT | Sequencing |  |
| TDRKH | TDRKH nested F | TATGTAGGGAGATGGTATG | First round nested PCR forward | 50 |
| TDRKH | TDRKH nested R | AAATCCCTACCCAAACTTAAC | First round nested PCR Reverse | 50 |
| WNT7B | WNT7B F | GATGGATAGGAAGTTAAGGGATAAG | Forward | 55 |
| WNT7B | WNT7B R | [Btn]ACCCCTAACCCCCCTATAACCC | Reverse | 55 |
| WNT7B | WNT7B S | AGAGAGAGAGGATTTATTTTTTTA | Sequencing |  |
| WNT7B | WNT7B nested F | GTTGAATTATGAGTGGTAT | First round nested PCR forward | 48 |
| WNT7B | WNT7B nested R | CTCATCACCTACCAAATATTAT | First round nested PCR reverse | 48 |

**Table S2.** Antibodies and dilutions used for western blotting experiments.

| **Protein targeted** | **Source** | **Clone/Cat#** | **Dilution** |
| --- | --- | --- | --- |
| Argpyrimidine | Oya et al. JBC 1999 | mAb6B | 1/10 000 |
| Alpha-tubulin | Sigma | mAb B-5-1-2 | 1/10 000 |
| Beta-actin | Sigma | A5441 | 1/10 000 |
| DNMT1 | Cell Signaling Technology | #5023 | 1/1000 |
| DNMT3A | Cell Signaling Technology | #3598 | 1/1000 |
| DNMT3B | Cell Signaling Technology | #67259 | 1/1000 |
| GLO1 | BioMAC (Leipzig, Germany) | mAb 4C10/#02-14 | 1/1000 |

**Table S3:** Primer sequences and probes used for RT-QPCR experiments.

| **Name** | **Foward / Reverse sequences** | **Probe (UPL, Roche)** |
| --- | --- | --- |
| 18S | CTTCCACAGGAGGCCTACAC / CGCAAAATATGCTGGAACTTT | #46 |
| ALCAM | GGCAGTGGAAGCGTCATAA / CATTCTCTTCAGGGGAAATGA | #6 |
| CDH1 | CCCGGGACAACGTTTATTAC / GCTGGCTCAAGTCAAAGTCC | #35 |
| CST6 | AGACACGCACATCATCAAGG/TCTCCAGTGACCCTGGTCTT | #21 |
| FST | TCCACTTTCAACAAACTTGCAT / AAAACCCGAGGGATCATGT | #27 |
| GULP1 | AACAAACATGTTACTATTCATTGGACA / GAAATTAGTGACCTTGTGGCATT | #48 |
| IFI30 | CTACGGAAACGCACAGGAA / CCATGCTGGCACTTGAACT | #59 |
| INPP4B | AACATATTGAACACAAGGCATCA / TCATTCTTTTTGGTCGTGCAT | #78 |
| KRT18 | TGATGACACCAATATCACACGA / ATCTGGGCTTGTAGGCCTTT | #17 |
| MUC1 | CAACTTGTAGGGGCACGTC / CCCTGAAGAACCTGAGTGGA | #20 |
| PSG1 | GAAGAGGCTCAGCACAGAAAG / GGAGATAAGCCTAGGATCCAGAA | #56 |
| RAB17 | TTCTAGCTGACAGTGGGAGGA / TGGAATCTTTGTTGCACTTGA | #26 |
| RBM47 | CCACTCCCACATCTCTCACC / TCCTAGGCTTGCCAAACATT | #52 |
| RHOBTB3 | GGGTAGTGCTTTGTGCTGGT / TCTTCTCTAATTAAAATGACTGGGAAA | #38 |
| SCNN1A | TGTGACTACAGAAAGCACAGTTCC / CCAGGTGGTCTGAGGAGAAGT | #1 |
| SERPINB1 | TGAGAATCTCGATTTCATTGAAGTTA / CGAGGTCGGAGTTGAGAGTG | #61 |
| TET1 | ACCATCTGTTGTTGTGCCTCT/GCCTTTAAAACTTTGGGCTTC | #57 |
| TET2 | CGACTGCAACTGCTGGATT/ACAAGGCTGCCCTCTAGTTG | #30 |
| TET3 | CGCCTCTATCCGGGAACT/CTTCCCCGTGTAGATGACCT | #25 |
| TSPAN8 | TGCCATTCCCTTCACTTGAT / GCACTGGGCCAGGATATTTA | #16 |

**Methylglyoxal: a novel upstream regulator of DNA methylation**

Gaurav Dube^1†^, Assia Tiamiou^2†^, Martin Bizet^1^, Yasmine Boumahd^2^, Imène Gasmi^2^, Rebekah Crake^2^, Justine Bellier^2^, Marie-Julie Nokin^2^, Emilie Calonne^1^, Rachel Deplus^1^, Tom Wissocq^2^, Olivier Peulen^2^, Vincent Castronovo^2^, François Fuks^1,3‡^ and Akeila Bellahcène^2‡*^

Data set description:

**Data S1:** Differentially Methylated CpGs and genomic sites in GLO1-depleted MDA-MB-231 cells.

**Data S2:** GSEA pathway analysis results.

**Data S3:** Hypermethylated and down expressed genes.

**Data S4:** Hypomethylated and overexpressed genes.

**Data S5:** Gene Body hypermethylated and overexpressed genes.

**Data S6:** Gene Body hypomethylation of down regulated genes.

**Data S7:** Expression-methylation status of 60 genes of MG signature.

**Data S8:** MG stress status of METABRIC cohort patients.

**Data S9:** MG stress status of TCGA cohort patients.
