# Supplementary figures and images for: Methylglyoxal: a novel upstream regulator of DNA methylation
Source: J Exp Clin Cancer Res. 2023 Mar 31;42:78. doi: 10.1186/s13046-023-02637-w (PMC10064647; doi:10.1186/s13046-023-02637-w)

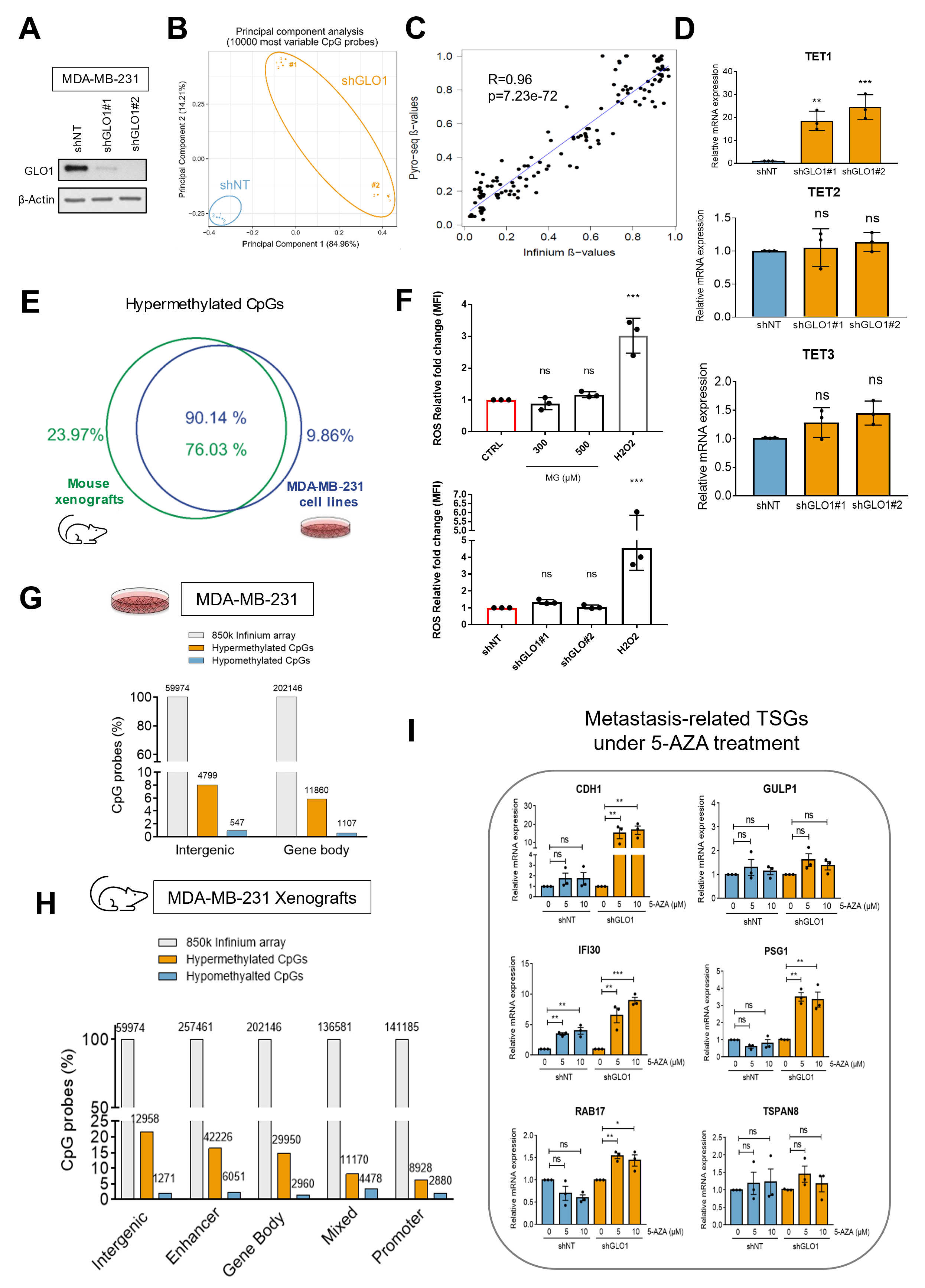

Supplement: Supplementary file 1 — Additional file 1. [file 13046_2023_2637_MOESM1_ESM.zip › Supplementary_Figure_S1.tif]

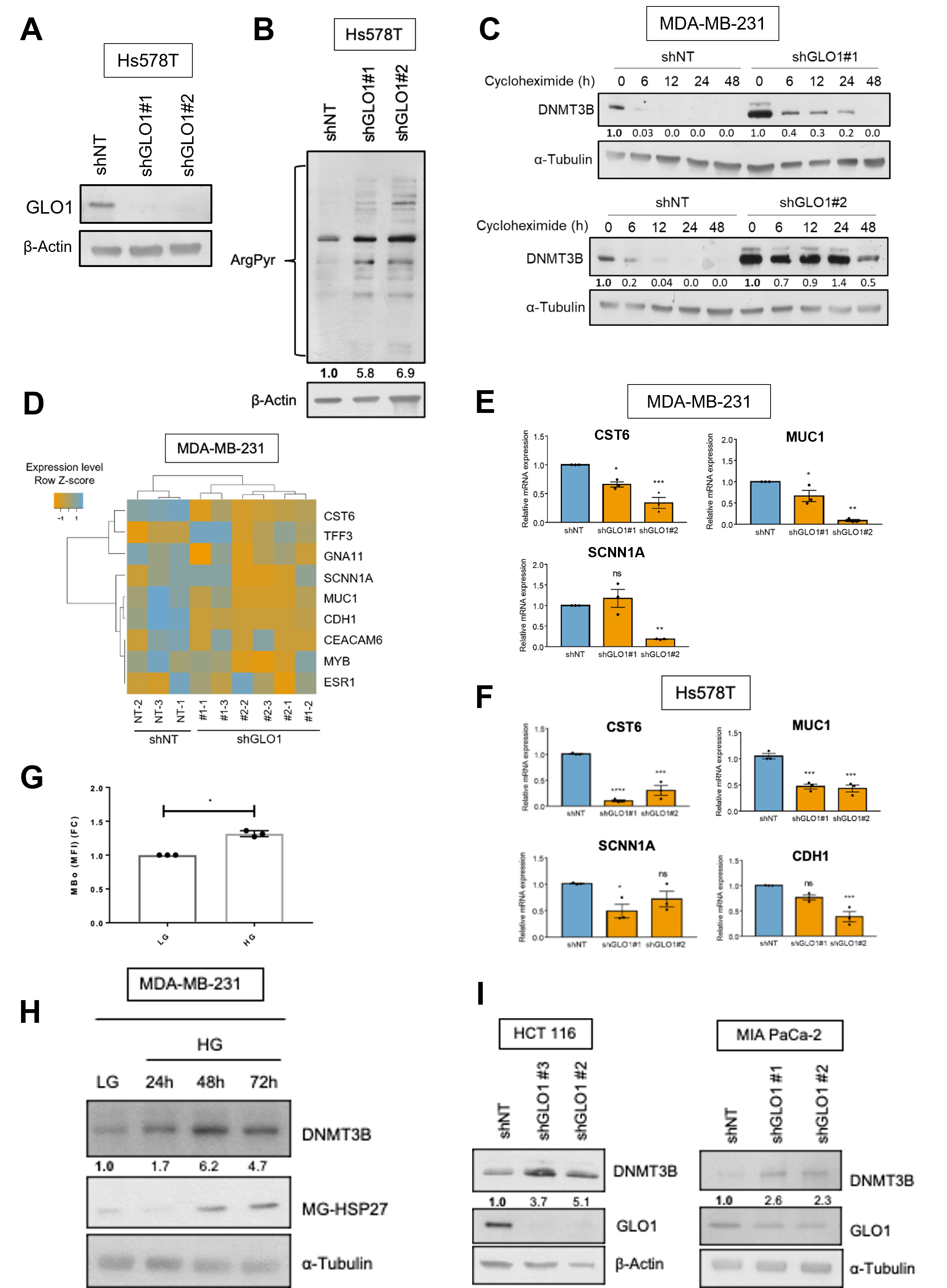

Supplement: Supplementary file 1 — Additional file 1. [file 13046_2023_2637_MOESM1_ESM.zip › Supplementary_Figure_S2.tif]

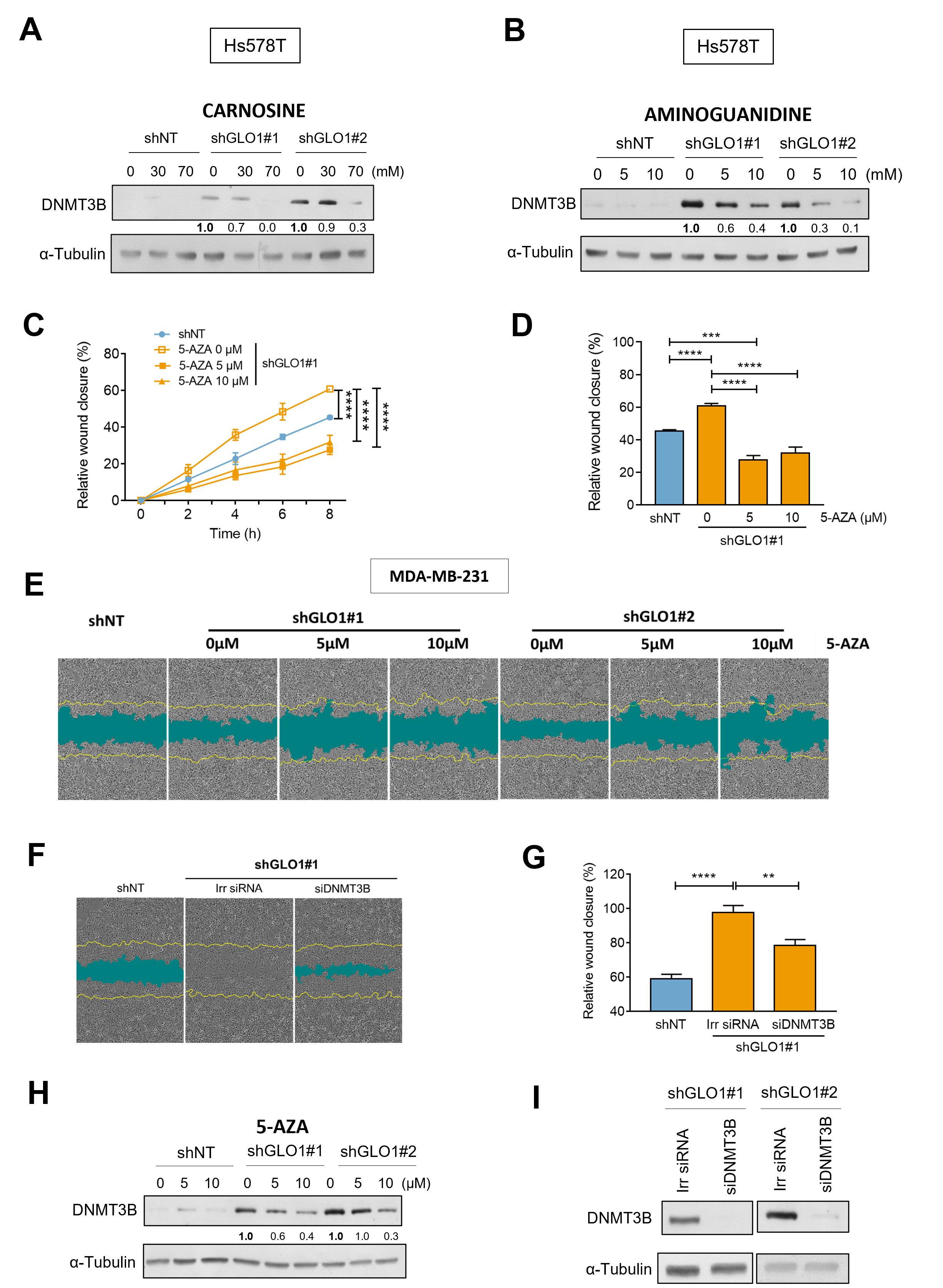

Supplement: Supplementary file 1 — Additional file 1. [file 13046_2023_2637_MOESM1_ESM.zip › Supplementary_Figure_S3.tif]

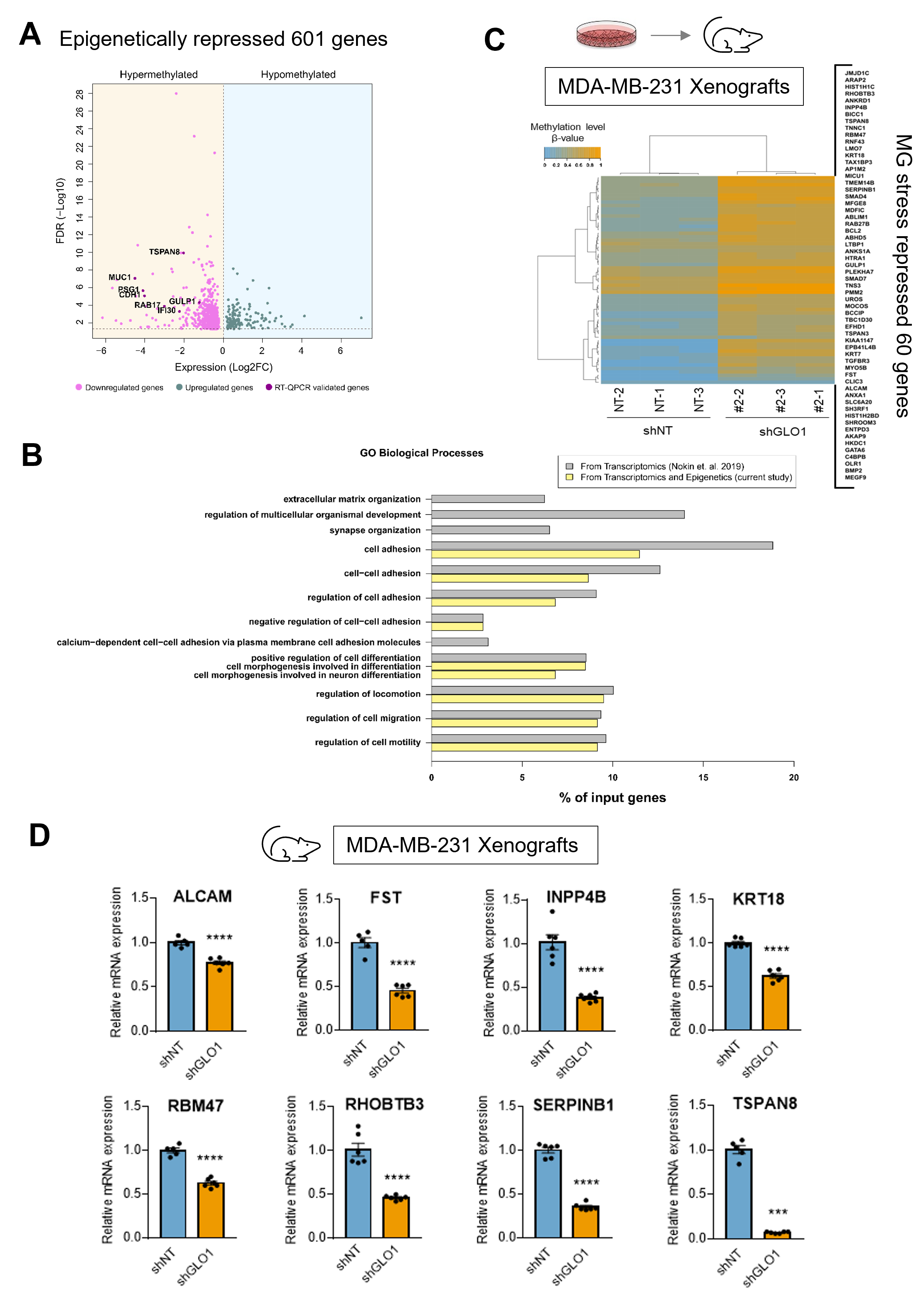

Supplement: Supplementary file 1 — Additional file 1. [file 13046_2023_2637_MOESM1_ESM.zip › Supplementary_Figure_S4.tif]

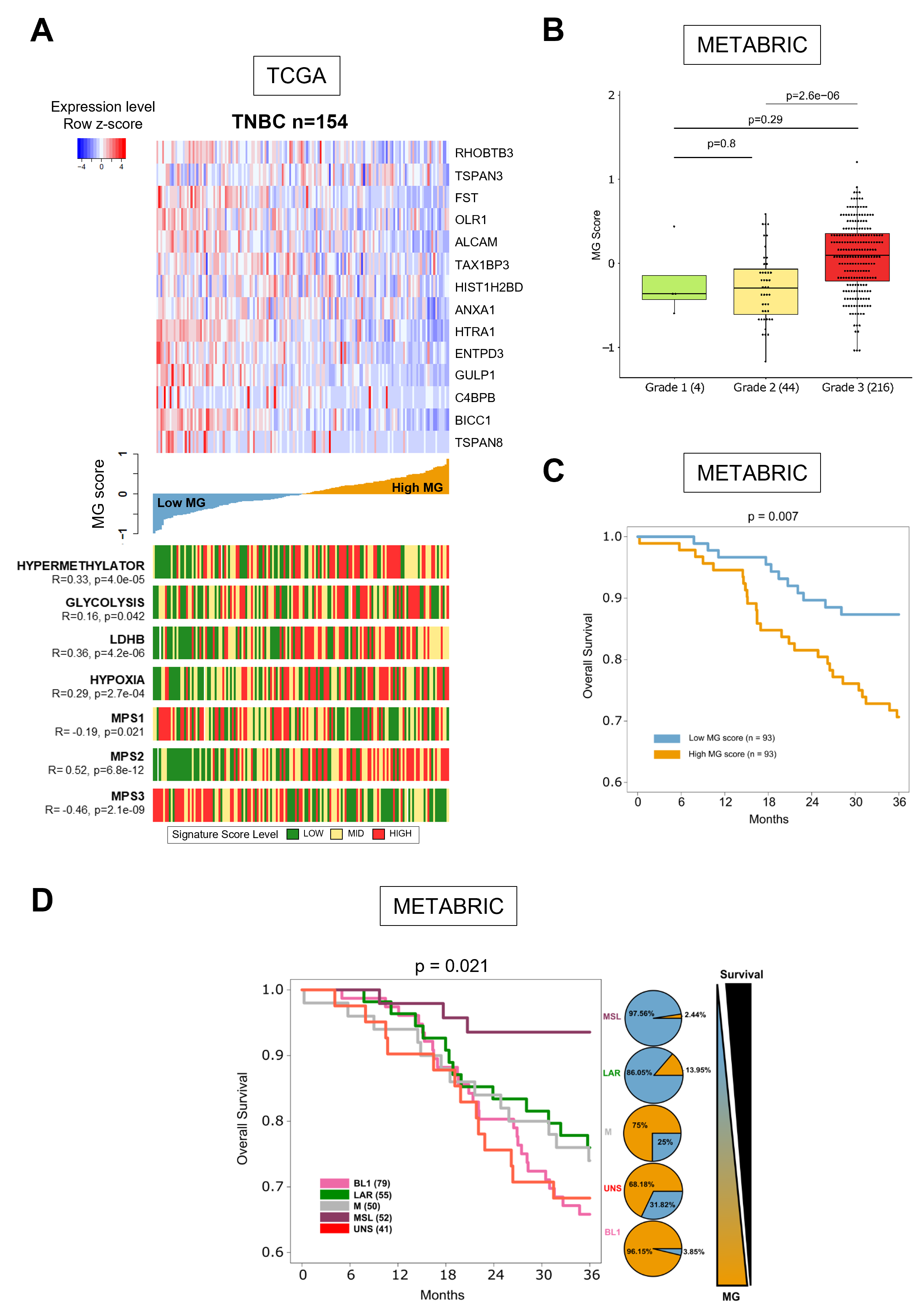

Supplement: Supplementary file 1 — Additional file 1. [file 13046_2023_2637_MOESM1_ESM.zip › Supplementary_Figure_S5.tif]
